# The critical issue linking lipids and inflammation: Clinical utility of stopping oxidative stress

**DOI:** 10.3389/fcvm.2022.1042729

**Published:** 2022-11-09

**Authors:** Bradley Field Bale, Amy Lynn Doneen, Pierre P. Leimgruber, David John Vigerust

**Affiliations:** ^1^Department of Medical Education and Clinical Sciences, Washington State University College of Medicine, Spokane, WA, United States; ^2^Department of Medical Education and Clinical Sciences, University of Washington School of Medicine, Seattle, WA, United States; ^3^Department of Neurological Surgery, Vanderbilt University School of Medicine, Nashville, TN, United States

**Keywords:** oxidative stress, platelet-derived growth factor, smooth muscle cell transformation, proteoglycans, remnant cholesterol, lipoprotein retention

## Abstract

The formation of an atheroma begins when lipoproteins become trapped in the intima. Entrapped lipoproteins become oxidized and activate the innate immune system. This immunity represents the primary association between lipids and inflammation. When the trapping continues, the link between lipids and inflammation becomes chronic and detrimental, resulting in atherosclerosis. When entrapment ceases, the association between lipids and inflammation is temporary and healthy, and the atherogenic process halts. Therefore, the link between lipids and inflammation depends upon lipoprotein retention in the intima. The entrapment is due to electrostatic forces uniting apolipoprotein B to polysaccharide chains on intimal proteoglycans. The genetic transformation of contractile smooth muscle cells in the media into migratory secretory smooth muscle cells produces the intimal proteoglycans. The protein, platelet-derived growth factor produced by activated platelets, is the primary stimulus for this genetic change. Oxidative stress is the main stimulus to activate platelets. Therefore, minimizing oxidative stress would significantly reduce the retention of lipoproteins. Less entrapment decreases the association between lipids and inflammation. More importantly, it would halt atherogenesis. This review will analyze oxidative stress as the critical link between lipids, inflammation, and the pathogenesis of atherosclerosis. Through this perspective, we will discuss stopping oxidative stress to disrupt a harmful association between lipids and inflammation. Numerous therapeutic options will be discussed to mitigate oxidative stress. This paper will add a new meaning to the Morse code distress signal SOS-stopping oxidative stress.

## Introduction

The two special issues of Frontiers in Cardiovascular Medicine devoted to the association of lipids and inflammation are significant collections in consideration of atherosclerosis. Arterial diseases, both macro and micro, represent the most prevalent health issues related to morbidity and mortality. Macrovascular disease leads to the most common cause of death and sustained disability. Heart attacks have remained the number one cause of mortality in the United States for over a century. Ischemic strokes are the fifth leading cause of death and the most common cause of severe long-term disability (1). Microvascular disease is prevalent and emerging as the primary etiology of chronic diseases of aging, including dementia (2). Such disease is responsible for Americans outliving their health by 13 years (3). The underlying pathology for both types of vascular disease is atherosclerosis (4). Pathogenesis of arterial disease depends on the development of chronic inflammation created by sustained activation of immunity from lipids (5). This review will discuss reducing oxidative stress as a measure to make this chronic situation addressable and surmountable.

## Background

The pathogenesis of atherosclerosis begins with trapping lipoproteins in the intima during extravasation from the arterial lumen (6). Lipoproteins up to 70 nm can pass through endothelial cells *via* transcytosis. Therefore, all lipoproteins except the largest very-low-density lipoproteins (VLDL) and chylomicrons can enter the arterial wall from the lumen in a healthy state. These particles diffuse through the wall and efflux on the adventitial side. The critical factor in forming an atheroma is the retention of migrating lipoproteins in the intima. This entrapment occurs *via* electrostatic forces between apolipoprotein B (apoB) and polysaccharide chains in proteoglycans (7). The captured lipoproteins become aggregated and oxidized.

The resulting ox-LDL activates the immune system stimulating endothelial dysfunction (ED). A critical component of the pathogenesis of atherosclerosis is ED (8). The ox-LDL stimulates innate immunity in endothelial cells through toll-like receptors (TLRs). Stimulation of TLRs results in intracellular chemical reactions which activate nuclear factor-kappa B (NF-kB). As a result, the cell nucleus produces inflammatory cytokines such as interleukin 1 beta, interleukin 6, interleukin 18, and tumor necrosis factor alpha. In addition, NF-kB increases the inflammasome nucleotide-binding oligomerization domain (NOD)-like receptor protein 3 (NLRP3). One crucial function of NLRP3 is the assembly of caspase 1. The cytokines IL-1 beta and IL-18 require cleavage by caspase-1 to become functional. There is an accompanying increase in mitochondrial reactive oxygen species (ROS) which has a bidirectional relationship with NLRP3. The mitochondrial and inflammasome activity increases and preserves the OS. These multiple reactions of inflammation contribute significantly to ED (9). Lectin-like receptors for ox-LDL in the endothelium upregulate the production of intercellular cell adhesion molecule-1 and vascular cell adhesion molecule, which recruit monocytes into the intima. The lipid-proteoglycan complexes get phagocytized *via* scavenger receptors, leading to foam cell formation (10). The foam cells will coalesce into fatty streaks and subsequent atheroma. Accumulating evidence indicates endothelial shear stress and autophagy also play a critical role in the endothelial dysfunction of atherosclerosis (11). Regardless of the evolving science about the mechanisms of ED, continued retention of lipoproteins will perpetuate ED leading to chronic inflammation and ongoing pathogenesis of atherosclerosis (12).

Preventing and halting atherosclerosis requires eliminating apoB and its associated LDL-C from getting trapped in the intima. The retention would cease if proteoglycans were absent within the intima. Proteoglycans originate from contractile smooth muscle cells (cSMCs) in the media, which get genetically transformed into migratory secretory smooth muscle cells (msSMCs). The msSMCs then move into the deep layer of the intima and produce proteoglycans (13). There are numerous triggers for the formation of msSMCs, but the most prominent is platelet-derived growth factor (PDGF). Therefore, inhibiting PDGF could significantly reduce atherogenesis (14). Oxidative stress (OS) is the primary stimulus to activate platelets (15). Activated platelets produce PDGF (16). Therefore, minimizing OS would significantly reduce the trapping of apolipoprotein B in the intima. Decreasing this first step in forming an atheroma would mitigate the association between lipids and chronic inflammation and impair the pathogenesis of atherosclerosis.

OS occurs when reactive oxygen species (ROS) are abundant relative to antioxidants. Mild OS can be beneficial. However, moderately or severely elevated levels of oxidants damage cellular components such as proteins, lipids, and nucleic acids. Such injury impairs cellular function and may cause apoptosis (17). Adenosine triphosphate (ATP), the primary cellular energy source, is formed from oxidant reactions. Mitochondria produce the majority of ATP and ~90% of ROS. Other cellular elements, including cytoplasm, cell membrane, endoplasmic reticulum, and peroxisome, generate a minor amount of ROS. To maintain homeostasis, all of these cellular elements generate antioxidants (18). The mechanisms responsible for ROS production are complex, as are the mechanisms for antioxidant formation, and the explanatory science is still evolving (19, 20). Despite this, the science is definitive that OS is a significant facilitator of chronic degenerative pathologies (21). Mitochondrial therapy is being investigated for many chronic disease pathologies, including atherosclerosis, due to the pivotal role of mitochondria in OS (22, 23).

This review will analyze OS as the critical link between lipids, inflammation, and atherosclerosis. CV risk factors, both known and emerging, will be examined through the lens of OS. The discussion will be about managing these conditions and the judicial use of therapeutic options to stop OS. Reducing OS would result in sustainable prevention and halting of arterial disease. This paper will examine results from a real-world clinic using such a comprehensive approach. Stopping OS (SOS) is paramount in breaking the association between lipids and chronic inflammation. SOS is vital in achieving arterial wellness.

## Traditional and emerging CV risks

In this section, known and evolving CV risk factors will be addressed through the lens of causing OS prior to the OS generated by lipoproteins retained in the intima. Any risk factor that produces such OS will enhance the trapping of lipoproteins and increase the chance of a chronic association between lipids and inflammation. This chronicity is due to an ongoing atherogenic process. Adequate management of CV risk factors will assuage OS, decreasing PDGF. As a result, msSMCs production will decrease, yielding the opportunity to halt atherogenesis (24).

### Lipoproteins

Lipoproteins are a well-known traditional CV risk factor. Low-density-lipoprotein -cholesterol (LDL-C) has garnered the most attention as a risk for arterial disease. However, decades of evidence indicate that apoB is more predictive than LDL-C (25). This review focuses on OS generation prior to lipoprotein trapping in the intima. Some lipoproteins can generate OS in the plasma natively before intimal oxidation. Very low-density lipoprotein (VLDL), intermediate-density lipoprotein (IDL), and chylomicrons are inflammatory in their indigenous state. In contrast, low-density lipoprotein (LDL) is not inflammatory until it becomes trapped and oxidized in the intima (26).

The explanation involves capturing VLDL, IDL, and chylomicrons on the endothelium. After attaching to the endothelium, lipoprotein lipase (LPL) hydrolyses them, releasing free fatty acids into the circulation (27). Free fatty acids activate systemic OS (28, 29). In addition, Shin et al. have shown that VLDL, IDL, and chylomicrons in the plasma generate OS *via* stimulating nicotinamide adenine dinucleotide phosphate (NADPH) oxidase-dependent superoxide formation (30). Therefore, decreasing remnant cholesterol (VLDL, IDL, chylomicrons) should mitigate the formation of PDGF, reducing the genetic transformation of cSMCs. Thus, decreasing the first step in the pathogenesis of atherosclerosis. Supporting evidence for this is accumulating. In 2020, Castañer et al. demonstrated that remnant cholesterol, but not LDL-C, was associated with major adverse cardiovascular events independent of other risk factors (31). A review paper argues that remnant cholesterol promotes atherosclerosis beyond LDL-C (32). A cohort-based study found that elevated remnant cholesterol significantly increased the risk of myocardial infarction, ischemic stroke, and peripheral artery disease (33). Remnant cholesterol rises significantly postprandially. Not surprisingly, postprandial lipidemia is associated with the development of atherosclerosis and a heightened risk for CV events. OS increases with this type of lipidemia (34, 35). Fortunately, the severity of remnant cholesterol can be reduced in numerous ways, from lifestyle to altering the gut microbiome to nutraceutical and pharmacological interventions (36, 37). Current evidence indicates a need to take a clinical focus on reducing remnant cholesterol.

### Hypertension

Hypertension is a significant risk factor for CVD. It is prevalent in approximately half of all Americans and at least a third of the world population (1). Hypertension was the number one risk factor for stroke (38). OS is central in hypertension. Factors involved in hypertension, such as angiotensin II, aldosterone, and endothelin 1, activate NADPH. This activity causes an increase in ROS, leading to systemic OS (39). As demonstrated in hypertension, the ensuing OS will stimulate cSMCs to transform into msSMCs (40). Managing hypertension can disrupt this detrimental situation and reduce CV risk (41). More intensive control can potentially lower CV risk further (42). Numerous interventions to treat hypertension include lifestyle, behavioral therapies, over-the-counter supplements, and pharmaceutical agents (43, 44). Globally, ~90% of patients with hypertension can be controlled (45). Achieving effective blood pressure management is a vital measure to prevent a chronic association between lipids and inflammation and the pathogenesis of atherosclerosis.

### Direct or indirect smoking

Direct or indirect smoking is the most documented modifiable risk factor for arterial disease. Smoking now includes hookahs, e-cigarettes, and cigarettes. The extracts from the smoke generate the CV risk. These particles generate OS by reducing mitochondrial deacetylase sirtuin 3 (SIRT3), which increases the hyperacetylation of superoxide dismutase (SOD). This reduces the clearance of reactive oxygen species (ROS), generating systemic OS. In addition, nicotine is a ligand for alpha1 nicotinic acetylcholine receptors, which also induces cSMCs to transform into msSMCs (46). Therefore, exposure to nicotine in any form, including patches, chewing, and passive or active smoking, initiates atherosclerosis's pathogenesis. Smoking cessation and nicotine exposure avoidance are critical in disrupting the pathogenesis of atherosclerosis and a chronic association between lipids and inflammation.

As expected, decreasing smoking reduces CV mortality in CVD patients (35). Many studies have evaluated interventions to assist in the discontinuation of smoking. One of the early ones showed the benefit of the “5A's”: ask, advise, assess, assist and arrange (47). Graphic pictures of the severe health issues from smoking can be a practical, motivational tool for smoking cessation (48). Evaluation of other factors to stimulate cessation have been published (49). More recently, a telemedicine approach using holistic counseling effectively enrolled non-motivated patients into treatment for cessation (50). Preventing atherosclerosis and a chronic association between lipids and inflammation demands smoking cessation.

### Type 2 diabetes

Type 2 diabetes (T2DM) is an undisputable CV risk factor and is generally considered a coronary artery disease equivalent (51). Insulin resistance (IR) is the main underlying issue that drives individuals to T2DM (52). IR is prevalent globally in all social and ethnic demographics (53). Approximately one-third of Americans are prediabetic with underlying IR (54). There is a decrease in peripheral tissue cellular response to insulin with IR. Understanding the mechanism causing the blunted response relates to the pathogenesis of arterial disease. OS is the critical mechanism for the pathogenesis of IR. Individuals at risk for new-onset diabetes and, thus, who are prediabetic, have underlying conditions that cause OS. Individuals may have one or many of these issues. They include the following: inadequate sleep (55), low vitamin D (56), autoimmune disease (57), periodontal disease (58), elevated remnant cholesterol (59), hypertension (60), psychosocial issues (61), chronic infectious disease (62), air pollution (63), nicotine exposure (64), gut dysbiosis (65), elevated myeloperoxidase (66), poor diet (67), and a sedentary lifestyle (68). The increased ROS produced by these conditions blocks the skeletal muscle and adipose cells' insulin receptor ability to phosphorylate insulin receptor substrate-1 (IRS-1). The non-phosphorylation of IRS-1 results in the failure of glucose transporter 4 (GLUT4) migration to the cell membrane. GLUT4 is critical for glucose uptake by the cell; without it, hyperglycemia ensues (69). If the IR continues, the pancreatic beta cells become fatigued, leading to hypoinsulinemia and diabetic hyperglycemia (70). As the core reason for IR, OS explains the strong association between T2DM and CV risk. With comprehensive care managing all the modifiable conditions mentioned above, IR is preventable. Avoiding IR is a monumental step toward reducing CV risk.

### Diet

Diet is well established as a factor influencing CV risk. Multiple healthy eating patterns are available (71). When evaluating various diets from an oxidative stress perspective, it is reasonable to expect a benefit from many diet patterns so long as the diet contains healthy choices. Foods that reduce CV risk contain significant amounts of antioxidants such as ascorbic acid, tocopherols, beta-carotene, flavonoids, polyphenols, and lycopene. Such natural antioxidant products include fruits, vegetables, grains, legumes, tea, and some fish. Consumption of these foods prevents or reverses systemic OS (72). This effect decreases PDGF production, reducing cSMCs transformation into msSMCs. Healthy foods decrease SMC transformation, which explains some of their anti-atherosclerosis effects (14). The amount of food eaten, even with healthy choices, is important. Overconsumption can lead to obesity and OS. Restricting calories by as little as 15% decreases OS (73). Remnant cholesterol surges postprandially. Therefore, it is no surprise that restricting consumption to a time frame of 10 h or less each day reduces systemic OS (74). The personalizing of the diet based on genetics is gaining traction as a clinical tool to influence circulating lipoprotein levels. Pérez-Beltrán et al. published an extensive review regarding various gene variants that influence diets and the resultant circulating lipid levels. Their publication summarizes dietary recommendations related to numerous genetic polymorphisms (75). A nutrigenetic panel can provide individualized dietary advice to lower triglycerides, which represent the inflammatory remnant cholesterol lipoproteins. Dietary compliance would reduce OS. Books will continue to be written on one size fits all panacea CV risk-reducing diets. Knowledge does not support such a diet. Science does support food can reduce CV risk. The choices need to be healthy antioxidant foods, quantities that do not increase weight, consumption during a daily 10-h or less time frame, and personalized with genetics.

### Physical activity

Physical activity is an irrefutable CV risk factor. Papers are continually published demonstrating either increased CV risk from being sedentary or decreased CV risk from being active (76). This finding is congruent with the effect of activity on OS. Chronic ROS overproduction in skeletal muscle cells induced by physical inactivity leads to systemic OS (77). Aerobic exercise reduces mitochondrial ROS production by suppressing NADPH oxidase expression. In addition, with aerobic exercise, there is increased catalase antioxidant activity. Damaged mitochondrial DNA (mtDNA) causes an increase in ROS. Exercise prevents the deterioration of mtDNA by increasing mtDNA content, enhancing ATP formation, and reducing mitochondrial swelling. It is important to note that resistive exercise reduces endothelin-1 (ET-1), an additional trigger for transforming cSMCs into msSMCs (78). Resistive exercise also significantly reduces OS. Elderly individuals who enrolled in a 12-week program demonstrated this effect.

The degree of physical activity is directly related to CV risk at any age (79, 80). As a critical initiator of atherogenesis, OS is fundamental in the relationship between activity and CV risk. Addressing physical activity with patients is a core element of management to reduce the risk of atherosclerosis. In the United States, guidelines recommend for adults 150 min a week of moderate-intensity or 75 min a week of vigorous-intensity. Only two-thirds of Americans are achieving those goals. In addition, the sedentary time has increased to 6.4 h a day (81). Latin Americans have similar guidelines recommending 150 min a week of moderate activity and <8 h/d of sedentary behavior. Only 48 and 22% achieved those goals, respectively (82). The Secretary of Health and Human Services issued a scientific report in 2018 on the vital importance of physical activity. The document contains a wealth of suggestions to assist in attaining higher compliance with physical activity (https://health.gov/our-work/nutrition-physical-activity/physical-activity-guidelines/current-guidelines/scientific-report). Educating people about the significance of avoiding OS to achieve arterial wellness could enhance conformity to guidelines. Physical activity is a powerful force to mitigate the pathogenesis of atherosclerosis.

### Weight

Weight, as indicated by BMI, is considered one of the eight essentials for heart health (83). Khan et al. demonstrated that female individuals with BMI >25 have increased CVD risk (84). All obese (BMI ≥ 30) people, regardless of metabolic health, have elevated CV risk (85). Obesity is a substantial issue in America, where ~40% of adults are obese [National Center for Health Statistics, 2018. National Health and Nutrition Examination Survey. 2012. URL: http://www.CDC.gov/nchs/nhanes.htm (accessed 2014-09-08) (WebCite Cache)]. The CV risk involves OS. There are multiple mechanisms generating the OS. Fat accumulation increases NADPH oxidase activity and endoplasmic reticulum stress in adipocytes leading to increased production of ROS. Obesity also decreased the activity of superoxide dismutase (SOD), which is a powerful antioxidant. Obesity is associated with increased leptin, remnant cholesterol, and blood glucose, all of which drive OS (86). Weight is modifiable through numerous measures, including counseling, exercise, diet, medications, and surgery (87, 88). Eliminating excess weight is an effective tool to subdue the pathogenesis of arterial disease and a chronic association between lipids and inflammation.

### Hyperuricemia

Hyperuricemia has been associated with CVD for decades. Numerous known CV risk factors, such as hypertension, abdominal obesity, insulin resistance, metabolic syndrome, and chronic kidney disease, are also associated with elevated uric acid. After adjusting for these known risk factors, some studies indicate hyperuricemia is not an independent CV risk factor (89). However, evolving evidence supports elevated uric acid as an independent CV risk factor (90). Hyperuricemia is associated with OS, which could provide the mechanism for an independent relationship with CVD. Impaired excretion of uric acid is the cause of ~90% of hyperuricemia. Elevated uric acid levels stimulate nicotinamide adenine dinucleotide phosphate (NADPH) oxidase, which leads to increased ROS. The resulting OS upregulates xanthine oxidoreductase activity which generates additional OS. This subject is complicated since uric acid can also perform as an antioxidant. Uric acid scavenges for carbon-centered radicals and peroxyl radicals which reduces OS. Uric acid provides approximately half of the antioxidant activity in serum (91). Hyperuricemia also can inhibit PDGF- induced cSMC transformation to msSMCs (92). The ALL-HEART study presented by Dr. Isla Shelagh Mackenzie at the European Society of Cardiology Congress, Barcelona, Spain, on August 27, 2022, failed to show benefits in reducing CV risk with the xanthine oxidase inhibitor allopurinol in patients with ischemic heart disease. Due to the above conflicting information, it appears premature to consider hyperuricemia an independent CV risk factor.

### Periodontal disease

Periodontal disease is an emerging CV risk factor (93). The high-risk periodontal pathogens are causal to atherosclerosis (94). One of the mechanisms involved is increasing the transformation of cSMCs to msSMCs (95). Periodontal disease, in general, also increases OS. Sharma and colleagues demonstrated the importance of OS generated by periodontal disease in chronic kidney disease (96). Therapy for periodontal disease reduces the levels of OS biomarkers (97). Therefore, the treatment of periodontal disease would reduce the pathogenesis of atherosclerosis by decreasing the trapping of lipoproteins in the intima. Approximately 65 million adults in the United States (US) have periodontitis, and in over 6 million, it is severe periodontal disease (98). Chronic periodontitis occurs in 68% of US adults ≥ 65 years of age (99). Globally periodontitis is the sixth most prevalent disease. Worldwide ~11% of adults have severe periodontitis (100). Managing periodontal disease represents a substantial opportunity to mitigate the chronic association between lipids and inflammation and reduce arterial disease.

In addition to periodontitis, other chronic infections are considered CV risk factors. Hepatitis C viral infection (HCV) is associated with a higher incidence of carotid atherosclerosis than non-HCV individuals. After adjusting for known CV risk factors, HCV independently predicts coronary and cerebral atherosclerosis risk (101). Human immunodeficiency virus (HIV) also appears to increase the risk of atherosclerosis independently. This finding suggests HIV should be considered a CV risk factor (102). Many chronic viral and bacterial infections are associated with heightened CV risk. Numerous mechanisms are involved in the overproduction of ROS by chronic infections. Studies have established that chronic infections increase oxidative stress (103). One consequence is the enrichment of proteoglycans in the intima to trap lipoproteins. Evidence is evolving that effective treatment of the pathogen causing the chronic infection will reduce CV risk (104, 105). Chronic infection will continue to evolve as a CV risk factor. Effective treatment of the causal pathogens would be expected to reduce the chronic association between lipids and inflammation and reduce the risk of atherosclerosis.

### Obstructive sleep apnea

Obstructive Sleep Apnea (OSA) is a well-established risk factor for strokes, heart attacks (106), obesity, metabolic syndrome, insulin resistance (107), hypertension (108), atrial fibrillation (109), and heart failure (110). The incidence of OSA is significant, affecting nearly one billion people worldwide (111), and represents a significant public health concern (112). A formal diagnosis of OSA utilizes a sleep study called polysomnography which monitors heart rate and rhythm, lung, and brain activity, breathing patterns, arm, and leg movements, and blood oxygen levels while asleep. The Diagnostic and Statistical Manual of Mental Disorders' five criteria for diagnosing OSA must record at least five obstructive apneas or hypopneas per hour of sleep (AHI). The apnea severity index includes mild apnea AHI 5–15, moderate AHI 15–30, and severe AHI > 30 (113). Airway obstructions predispose airways to collapse during inspiration, leading to alveolar hypoventilation, which induces sleep fragmentation through central nervous system activations (arousals). This intermittent hypoxia causes alterations in metabolic and immune system changes (114). OSA (at all levels of severity) results in recurrent periods of hypoxemia followed by reoxygenation, leading to ROS overproduction and increasing the inflammatory response. Chronic episodes of hypoxia followed by subsequent reoxygenation lead to the impairment of mitochondrial oxidative phosphorylation and induce the production of ROS, causing OS. In response to the OS due to OSA, the cSMCs become msSMCs (115). Many emerging and well-established therapies are available to treat OSA. These include the traditional continuous positive airway pressure therapy (CPAP), oral appliance (OA), and even upper airway stimulation devices. Depending on the severity of the OSA, each of these treatments can lead to amelioration of OSA (116). Data continues to emerge regarding the effectiveness of these treatments on the milieu of oxidative stress caused by untreated OSA (117). Diagnosis and management of OSA is critical in decreasing the chronic association of lipids and inflammation as well as atherosclerosis.

### Psychosocial

Psychosocial is an established risk factor for cardiovascular risk. Epidemiological studies have confirmed the high co-morbidity between heart disease and psychosocial illnesses such as depression and anxiety. Immune system imbalance due to depression leads to platelet activation (118). Anxiety and depressive syndromes are associated with serotonin disruption. Serotonin is involved with the homeostasis of platelet activity, vascular tone, thrombosis, and vasoconstriction resulting in negative CV consequences (119). Depression changes oxidative stress-related enzymes' activity leading to oxidative stress (120). Chronic stress response activates the nervous and sympathetic nervous system, ultimately stimulating cortisol secretion, leading to the onset of inflammation and OS (121). Severe anxiety is associated with OS in major depressive disorders (122). Evidence suggests a bidirectional association between psychosocial disorders and OS (123). Anti-depressive therapy reduces biomarkers of oxidative stress (124). Educational stress management reduces OS (125). Clinically, diagnosing and managing psychosocial impairments is essential to reduce the chronic association between lipids and inflammation by mitigating atherosclerosis's pathogenesis.

### Vitamin D deficiency

Vitamin D deficiency is the most common nutrient deficiency worldwide. Low vitamin D levels are independently associated with coronary and carotid artery disease (126, 127). The inactive vitamin D precursors undergo 25-hydroxylation in the liver to form 25-hydroxyvitamin D [25(OH)D] (128). 25(OH)D is the primary circulating form of vitamin D and is therefore considered a circulating biomarker for vitamin D status. The definition of vitamin D insufficiency is serum 25(OH)D concentrations lower than 50 nmol/l (20 ng/ml) (129). Vitamin D is a potent antioxidant that facilitates healthy mitochondrial activity. Low levels of vitamin D impair mitochondrial functions, which causes OS. Adequate levels of vitamin D downregulate intracellular oxidative stress-related activities (130). Vitamin D has also been demonstrated to play a role in intracellular nuclear factor erythroid 2–related factor 2 (Nrf2) levels which are inversely related to the buildup of mitochondrial ROS, further leading to oxidative stress (131).

The knowledge about vitamin D and OS is congruent with the fact that vitamin D deficiency stimulates platelet activation (132). The activated platelets increase PDGF, increasing cSMCs to transform into msSMCs. A murine model showed that vitamin D supplementation inhibited the proliferation and transformation of vascular SMCs (133).

As expected, the increase in msSMCs will lead to retention of lipoproteins in the intima resulting in endothelial dysfunction through upregulation of NF-kB. Vitamin D decreases the signaling of NF-kB, which decreases the production of inflammatory cytokines (134). The cytokine IL-6 has a pro-atherothrombotic effect which vitamin D inhibits (135). Generally, vitamin D mitigates the harmful OS effects on the endothelium (136). Given the wealth of evidence that vitamin D deficiency is associated with numerous CV risk factors and the incidence of CVD, it is surprising that randomized trials have failed to show the benefit of vitamin D supplementation for CVD (137). However, these trails have numerous limitations. One of the most significant is that no trials selected participants based on being deficient in vitamin D. Other issues to include are lack of power to demonstrate CVD benefits, significant variations in dosage of the supplement, inadequate length of follow-up, inability to analyze individual subject data and lack of standardization of 25(OH)D assay (138). Considering the overwhelming evidence that vitamin D deficiency is a CV risk factor, it seems prudent to assess a patient's 25(OH)D level clinically. If the level is inadequate, it seems reasonable to recommend management that would create an adequate level. Such action may impede the pathogenesis of atherosclerosis and the chronic association between lipids and inflammation.

### The gut microbiome

The gut microbiome is considered the largest endocrine organ due to its immense production of biologically active metabolites. Adverse changes in the microbial composition are known as gut dysbiosis, which is associated with numerous CV risk factors, including hypertension, hyperlipidemia, and insulin resistance (139). Compared to individuals without atherosclerosis, patients with atherosclerosis have increased levels of *Streptococcus* and *Enterobacteriaceae* species (140). The gut microbiome signature represented by stool was significantly different in patients with coronary artery disease compared to healthy subjects. CAD patients had an abundance of *Escherichia-Shigella* and *Enterococcus* with a paucity of *Faecalibacterium, Subdoligranulum, Roseburia*, and *Eubacterium rectale* (141). Evidence continues to accumulate that gut dysbiosis is associated with the pathogenesis of atherosclerosis (142).

The gut microbiota metabolites influence mitochondrial ROS production, the production of antioxidants like glutathione, and Cyclooxygenase- 2 (COX-2) activity. These mechanisms allow commensal bacteria such as Lactobacilli and *Bifidobacteria* to lower OS and pathogenic bacteria like *Salmonella* and *E. coli* to increase OS (143–145). The relationship between systemic OS and gut dysbiosis is multidirectional. An increase in systemic OS increases the *E. coli* and *enterococcus* populations while lower the levels of *lactobacilli* (146). Mitigation of systemic OS with an antioxidant influenced the gut microbiome by increasing the quantity of commensal *Lactobacillus* and Bifidobacterium while decreasing the amount of pathogenic *E. coli* (147). The study of the gut microbiome is in its infancy. Scientists need to continue investigating the vastness of the role of gut microbial metabolites on health and OS. Therapies such as fecal transplants and probiotics need more formal research (148). The gut microbiome plays a critical role in CV health. There is a paucity of human data elucidating mechanistic insights. Future investigations are needed to improve clinical assessment and management of this novel CV risk factor (149).

### Autoimmune diseases

Autoimmune diseases are well established as associated with heightened CV risk. Rheumatoid arthritis (RA), systemic lupus erythematosus (SLE), psoriasis, Sjogren's syndrome, and Crohn's disease show an increased risk of CV disease (150). RA increases the risk of a heart attack 7-fold, making it on par with T2DM for CVD risk (151). SLE triples the risk of heart disease (152). A hallmark feature of autoimmune disease is the increased production of ROS and reactive nitrogen species (RNS), leading to OS. An essential source of elevated ROS is mitochondrial dysfunction. Patients with RA compared to healthy subjects have a five-fold increase in mitochondrial ROS production. In addition, autoimmune disease patients generally have depleted levels of the antioxidant glutathione (153).

Rheumatoid arthritis (RA) as a prototype for autoimmune disease has been studied extensively in terms of OS. Mitochondria generate most of the ROS, creating the OS, which has a central role in the pathogenesis of RA. The relationship between OS and mitochondria is bidirectional. ROS attacks mitochondria causing destabilization and mutations of mtDNA. The damaged mitochondria create increased levels of ROS, resulting in a vicious cycle of OS. This knowledge helps explain why tumor necrosis factor (TNF) blocking agents reduce CV in RA patients. These agents suppress mitochondrial-generated OS. It also reinforces the need to study natural antioxidant agents known to reduce OS due to mitochondria, such as omega-3 and resveratrol (154). Clinically, it is reasonable to believe that management of autoimmune diseases focused on reducing OS will retard the pathogenesis of atherosclerosis and decrease the chronic association between lipids and inflammation.

### Air pollution

Air pollution, a non-traditional CV risk factor, is exposure to fine particulate matter with a median aerodynamic diameter of < 2.5 micrometers (μm) (PM2.5). Long-term exposure below the Environmental Protection Agency standard of < 12 μg/m^3^/annually increases CV risk (155). A study done in the European Union concluded that the risk from air pollution was higher than expected and significantly increased CV mortality (156). PM2.5 contains ROS and generates ROS from redox-active components. The current science unequivocally indicates that air pollution increases OS (157). OS is the central pathophysiological mechanism by which PM2.5 induces CV risk (158). This mechanism is supported by Abohashem et al.'s paper discussing animal studies that demonstrate PM2.5 activates the immune system resulting in increased production of monocytes from bone marrow and spleen. These monocytes then enter the atheroma. Using 18F-fluorodeoxyglucose positron emission tomography/computed tomography (18F-FDG-PET/CT) in humans, they found a direct relationship between the degree of PM2.5 exposure and the degree of activity in the bone marrow and spleen. In addition, there was a direct relationship between the amount of PM2.5 exposure and inflammation in the ascending aorta. These results are expected consequences of trapping lipoproteins in the intima. The conclusion was that chronic PM2.5 exposure independently increases CV risk. They also mention that over 90% of the world population is exposed to levels of air pollution exceeding the WHO Air Quality Guidelines (AQG) of annual PM2.5 exposure of 10 μg/m^3^, or 25 μg/m^3^ 24-h mean (159). Clinically, altering a patient's habitat is challenging, but patients deserve education about the CV risk of air pollution (160). Government policies to reduce PM2.5 should be a high priority (161). Individuals can check daily reports on the current level of air pollution. If the levels are unsafe, they should do their best to breathe filtered air. Reducing exposure to PM2.5 would positively decrease the chronic association between lipids and inflammation and reduce the risk of arterial disease.

### Chronic kidney disease

Chronic kidney disease (CKD) is defined as two glomerular filtration rates (GFR) < 60 ml/min/1.73m^2^ during 3 months or more or confirmed kidney damage for at least 3 months. CKD is considered an independent risk factor for CVD (162). CVD mortality doubles in stage 3 CKD and triples in stage 4 CKD (163, 164). A hallmark of CKD is OS which is present in the initial stages of the disease. CKD is strongly associated with age, hypertension, insulin resistance, dyslipidemia, nicotine use, OSA, vitamin D deficiency, air pollution, gut dysbiosis, neuropsychiatric disorders, poor lifestyle, obesity, autoimmune disease, periodontal disease, chronic infection, and hyperuricemia (96, 165–176). It seems reasonable that one or more of these conditions that increase OS may initiate the onset of CKD.

The increase in ROS injures the glomeruli. The OS generates structural injury to the very susceptible podocytes leading to glomerulosclerosis. A critical factor in the progression of CKD is the activation of transforming growth factor beta (TGF-β) in the podocytes. TGF-β suppresses mitochondrial function leading to additional OS and subsequent damage to mitochondrial DNA. The mechanisms through which mitochondrial damage and cellular dysfunction promote progression to renal failure are still under investigation. The increased albumin resulting in the urine can enter cells in the proximal tubules. As a result, protein kinase C (PKC) is activated, which further increases OS *via* ROS produced by NADPH oxidase. As explained earlier in the paper, hyperuricemia due to CKD can also heighten the OS level. Asymmetrical dimethylarginine (ADMA) levels are elevated in CKD. ADMA inhibits nitric oxide (NO) synthesis. Less NO results in an increase in ROS (177).

**Figure 1:**
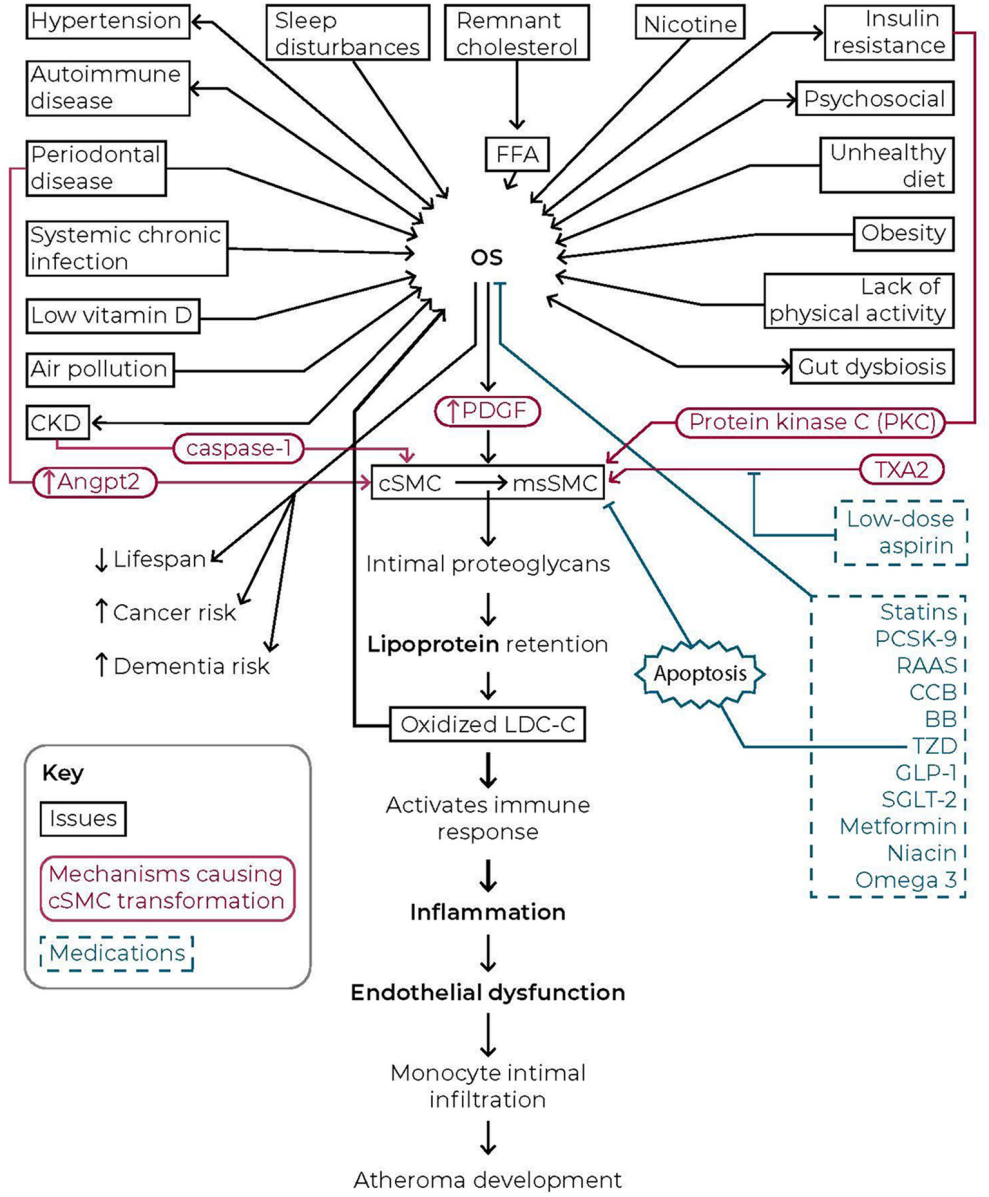
Comprehensive schematic of the role oxidative stress plays in cardiovascular disease. The multiplicity of risk factors for cardiovascular disease that drive oxidative stress which in turn results in a well-defined cascade of events that drive the development of atheroma in the vessel is presented. These cellular events lead to a decrease in lifespan and an increase in dementia and cancer risk. The linkage between lipids and inflammation is a critical consideration in the management of cardiovascular disease. Reduction in critical risk factors and the judicious use of medications (blue) can reduce levels of oxidative stress and in turn halt the progression of events leading to the development of atheroma.

CKD is associated with decreased levels of cSMCs with an increase in msSMCs. Initially, hyperuricemia was felt to be the stimulus (92). However, more recent evidence indicates that caspase-1 is essential in CKD to induce the phenotypic change in cSMCs. CKD produces danger signal-associated molecular patterns such as hyperuricemia which can stimulate the innate immune system through TLRs generating caspase-1. CKD initiates a switch in SMCs from cSMCs to msSMCs. The mechanisms for this remain to be fully elucidated (178). It is apparent that mitigating the chronic association between lipids and inflammation requires curtailing the incidence of CKD (see Figure 1).

## Treatment

Stopping oxidative stress (SOS) requires clinical management of all the discussed modifiable traditional and non-traditional CV risk factors. Those issues are remnant lipoproteins, hypertension, smoking, weight, diet, physical activity, insulin resistance, sleep, psychosocial, periodontal disease, other chronic infections, autoimmune disease, gut dysbiosis, vitamin D, and air pollution. This type of healthcare requires a holistic approach along with interdisciplinary collaboration. OS is the common denominator of the risk factors, making them interrelated (see Figure 1). For example, OS is at the root of insulin resistance pathogenesis which leads to T2DM. Therefore, it is no surprise that the above risk factors are associated with an increased risk of new-onset diabetes (55–64, 179–182). Accomplishing SOS demands comprehensive, integrated healthcare. Numerous frequently used drugs impact OS either directly or indirectly (183). This review analyzes many of these agents used to help manage CV risk through the lens of directly mitigating OS.

3-hydroxy-3-methylglutaryl coenzyme A (HMG-CoA) reductase inhibitors have evolved as a cornerstone medication to reduce CV risk. These drugs, better known as statins, are recommended in guidelines with benefits greatly outweighing harm (184). When examined through the perspective of OS, these agents excel. Within hours statins progressively reduce OS (185). Elevated NADPH-oxidase expression and activity create OS. A critical substance in the assembly and activity of NADPH-oxidase is Rac1, a guanosine triphosphatase. Stains decrease mevalonate, which results in a reduction in Rac1. The reduction in mevalonate causes a decrease in geranyl-geranyl-pyrophosphate (GGPP). Less GGPP causes increased expression of endothelial nitric oxide synthetase (eNOS). Statins help activate eNOS *via* phosphorylation. ENOS enhances the restoration of oxidative balance (186). As a significant bonus, statins increase the concentration of two powerful antioxidants, glutathione peroxidase and superoxide dismutase (187). This SOS effect of statins would reduce the PDGR-driven cSMC transformation. Chen and colleagues demonstrated this effect of statins (188). The biomarker lipoprotein-associated phospholipase A-2 (PLAC2) increases during the pathogenesis of atherosclerosis (189). The reduction in PLAC2 in the statin trail LIPID accounted for most of the statin benefit (190). It is conceivable that statins primarily mitigate CV risk *via* directly reducing OS.

Bempedoic acid (BA) lowers cholesterol in a similar manner to statins. It inhibits ATP-citrate lyase (ACL), a catalyst necessary to form acetyl-CoA. Less production of acetyl-CoA causes decreased formation of HMG-CoA. Thus, it acts upstream from statins to inhibit HMG-CoA production. Just like statins, this will decrease mevalonate formation, which should result in the activation of eNOS, helping to restore oxidative balance. BA theoretically has the potential to lower remnant cholesterol since acetyl-CoA activity can lead to increased TG (191). However, studies have shown no significant decrease in TG levels to date (192–195). BA has not been investigated for its ability to increase antioxidants. No data is available evaluating BA's effect on PLAC2. BA does carry a warning of increasing uric acid. There currently is no CV outcome data, albeit some is expected later this year with the CLEAR trial (Evaluation of Major Cardiovascular Events in Patients With, or at High Risk for, Cardiovascular Disease Who Are Statin Intolerant Treated with Bempedoic Acid). The medication requires prior authorization, and the cost is a concern (196). It remains to be determined if BA is an effective agent to reduce OS.

Because triglyceride (TG) reduction independent of LDL-C lowers CV risk, lipid-lowering agents beyond statins are frequently required (197). TGs are a surrogate representation of remnant cholesterol, which natively creates OS. Therefore, any therapy that lowers TG will directly lower OS. The question is; do any of these therapies directly reduce OS by other mechanisms? Fibrates decrease the progression of atherosclerosis and reduce CV risk (198). However, the evidence does not support that fibrates reduce OS beyond what would be expected from lowering TG (199). Numerous studies confirm that proprotein convertase subtilisin/kexin type 9 (PCSK9) inhibitors significantly lower TG and CV risk (200–202). In addition to PCSK9 inducing LDL receptor degradation, it activates NADPH oxidase promoting OS. Therefore, PSCK9-inhibitors (PCSK9-I) may reduce OS beyond reducing TG. Accumulating evidence suggests PCSK9-I may reduce CV risk *via* SOS (203).

Niacin, the water-soluble vitamin B3, is the oldest and one of the most effective agents to affect all lipoproteins favorably (204). In an *in vitro* study utilizing human aortic endothelial cells, niacin inhibited ROS production by angiotensin II (205). Seventeen subjects with an insulin resistance type dyslipidemia demonstrated that 2 grams of niacin a day for 3 months significantly reduced TG and OS (206). However, the degree of reduction in OS in that study may have been due to the lowering of TG. Niacin is a precursor for nicotinamide adenine dinucleotide (NAD^+^). Recently it was discovered that NAD^+^ is a co-substrate for the sirtuin family of deacetylases which help maintain mitochondrial homeostasis. The benefits of niacin may primarily be related to its influence on the NAD^+^/sirtuin-axis (207). *In vitro* and *in vivo* trials have confirmed that niacin reduces OS (208). Whether or not the mechanism for this reaches beyond its effect on TG is not established.

Omega 3 fatty acids (FAs) are an effective therapy for TG (209). A meta-analysis evaluating the effect of omega 3 FAs vs. placebo on the oxidative stress biomarker malondialdehyde (MDA) concluded a significant decrease in MDA with omega 3 FAs. The analysis also showed significantly increased activity of the antioxidant enzyme glutathione peroxidase (GPx). The conclusion was that omega 3 FAs reduce OS. It was noted in the analysis that the change in MDA seemed to be due to the improvement in the lipid profile (210). The evidence indicates that the benefit of SOS with omega 3 FAs is due to a reduction in TG. In summary, all TG-lowering agents SOS, but the only one that does so beyond the lipid effect, maybe PCSK9 inhibitors.

Evidence indicates that blood pressure medications reduce CV risk regardless of a hypertensive diagnosis. Healthcare providers should focus communication on the benefit of these agents on CV risk rather than the blood pressure benefit (211). Renin-angiotensin-aldosterone system (RAAS) medications have been considered a cornerstone for individuals with CVD (212). RAAS agents have pleiotropic effects beyond blood pressure reduction (213). RAAS therapy can double the antioxidant extracellular superoxide dismutase (EC-SOD) concentration. The change in EC-SOD partially explains the reduction in OS by RAAS medications (214). The main feature of RAAS agents is to reduce the effects on angiotensin II (AngII) either by reducing the production of AngII or blocking the receptor to activate AngII. AngII activates NADPH oxidase, which leads to increased ROS and OS (215). Amlodipine is a frequently used agent in individuals with CV risk. Amlodipine is known to reduce the progression of atherosclerosis. One mechanism by which it accomplishes this is inhibition of cSMC transformation and migration (216). Utilizing nitrotyrosine as a marker of OS, Kim and colleagues demonstrated therapy with amlodipine and a RAAS agent, and each significantly lowered the level after 24 weeks. The authors concluded that amlodipine reduces OS (217). Beta-blockers have a prominent role in managing CV risk. Carvedilol significantly reduced OS as measured by thiobarbituric acid-reactive substances (TBARS) (218). *In vitro* data have shown that carvedilol reduces ROS production by mitochondria (219). In a study of 44 patients with essential hypertension, multiple biomarkers, including the ferric-reducing ability of plasma (FRAP), glutathione/oxidized glutathione, malondialdehyde, and plasma 8-isoprostane, were used to assess OS. Twenty-three patients received carvedilol 12.5 mg/d, and twenty-one received nebivolol 5 mg/d for 12 weeks. All four biomarkers of OS improved significantly with carvedilol. There were no significant changes with nebivolol (220). These beneficial effects on the pathogenesis of atherosclerosis of the above three major classes of blood pressure medications were assessed in a comprehensive review of multiple strategies to manage hypertension (221). A significant reason the hypertensive medications amlodipine, carvedilol, and RAAS agents reduce CV risk is related to directly reducing OS.

Insulin resistance (IR), a CV risk factor, frequently requires medication therapy regardless of T2DM status. Hyperglycemia which results from IR, generates OS by multiple mechanisms (222). Therefore, any medication utilized to reduce IR and hyperglycemia will reduce OS. The question is, do any of them directly reduce OS by mechanisms other than reducing hyperglycemia. The alpha-glucosidase, dipeptidyl peptidase-4 inhibitors, and sulfonylurea agents have shown that they reduce OS (223–225). However, there is no evidence that the mechanism responsible reaches beyond reducing hyperglycemia. Metformin activates adenosine monophosphate-activated protein kinase (AMPK) (226). This activation leads to the expression of the antioxidant genes for the production of catalase (CAT), glutathione (GSH), and superoxide dismutase (SOD) (227). The increase in these agents enhances the antioxidant system, reducing ROS (228). Activation of AMPK also maintains the cSMC phenotype (229). The mechanism of activating AMPK will reduce the initial step in the pathogenesis of atherosclerosis and is a mechanism separate from reducing hyperglycemia. Thiazolidinediones (TZD), glucagon-like peptide-1s (GLP-1), and sodium-glucose cotransporter 2 inhibitors (SGLT-2) also activate AMPK (230–232). The TZD pioglitazone also destroys SMCs, which have transformed into msSMCs (233). These actions are a significant benefit in shutting down the pathogenesis of atherosclerosis. Indeed, pioglitazone has excellent studies showing it has a positive effect on mitigating the progression of atherosclerosis in IR patients regardless of diabetic status (234–237). In summary, all antidiabetic drugs SOS. Metformin, GLP-1, SGLT-2, and TZD agents do so by effects outside of mitigating hyperglycemia. The best medication for SOS appears to be pioglitazone with its unique ability to cause apoptosis of msSMCs.

## Discussion

A chronic association between lipids and inflammation develops with active pathogenesis of atherosclerosis. The first step in the pathogenesis involves trapping apolipoprotein B (apo B), which contains all the vascularly harmful cholesterol, in the intima. The contained cholesterol particles become oxidized and generate inflammation. The inflammation comes from activating the immune system to remove the oxidized particles from the arterial wall. The innate immune system initially responds by generating inflammatory cytokines and monocyte chemoattractant proteins affecting the endothelium. Subsequently, monocytes are captured on the endothelium and penetrate the intima. Macrophages are formed from those monocytes to digest the oxidized lipids. The adaptive immune system responds with effector T cells stimulated by antigens such as oxidized lipoproteins (238). If the trapping continues, the macrophages become engorged, becoming foam cells. In addition, SMCs may also become engorged with oxidized lipoproteins contributing to the mass of foam cells (239). If the process continues, the foam cells coalesce into fatty streaks and form atheroma. Active pathogenesis of atherosclerosis produces substances such as oxidized LDL and matrix metalloproteinase-2, which also contribute to the transformation of cSMCs (240). The three essential elements involved in the pathogenesis of atherosclerosis are apoB, endothelial permeability, and binding of lipoproteins in the intima (241). Individual analysis of these three components is helpful when attempting to end the chronic association between lipids and inflammation.

Would solely focusing on apoB stop the chronic association between lipids and inflammation? Excellent health requires cholesterol and fat. Cholesterol is a vital hormone ingredient, now recognized as an essential regulator of innate immunity (242). Fat is also an essential ingredient for health (243). Their transport to cells depends on apoB. Therefore, extreme reduction or elimination of this component is not feasible. It is only harmful when it gets trapped in the intima or if it contains high levels of remnant cholesterol. If there is no retention of apoB in the intima, an individual could have high levels with low CV risk. The converse would also be true. If there is the retention of apoB in the intima, an individual could have low levels with high CV risk. There is evidence congruent with retention being the critical determinant for arterial disease. One study found that about half of the 231,986 people with coronary artery disease had low levels of apoB (244). Studies also demonstrate that the absolute risk for atherosclerosis in many people with high apoB is low, albeit the relative risk might be twice that of subjects with low apoB (245). The main factor determining the number of lipoproteins retained in the intima is the number of proteoglycans available to bind apoB. The quantity of apoB diffusing through the arterial wall far exceeds the quantity trapped in the intima (7). Thus, reducing the plasma apo B concentration will decrease the intimal retention to some degree, but it will not prevent it. As long as there is the intimal binding of apoB regardless of apoB concentration, there will be a persistent association between lipids and inflammation.

Would focus on endothelial permeability and the responsible immune milieu end the chronic lipid inflammatory association? The immune activation and subsequent endothelial response are mechanisms to maintain arterial wellness. Interfering with this process might reduce inflammation enough to lower the risk of an underlying atheroma creating a thrombus from endothelial rupture or erosion. CV event risk in patients with known arterial disease was lowered with the medication canakinumab, which inhibits the proinflammatory cytokine interleukin-1β. Such action, however, does not halt the intimal binding of apoB. Continuation of the pathogenesis of atherosclerosis was evident, with a significant number of patients receiving canakinumab requiring hospitalization for unstable angina (246). The immune reaction and resultant inflammation are designed to restore wellness. Should the unhealthy retention of apoB continue, the chronic association between lipoproteins and inflammation will remain.

Stopping the binding of apoB in the intima would end the chronic association between lipids and inflammation. Proteoglycans have a protein core with negatively charged glycosaminoglycan chains. ApoB carries a positive charge. This electrostatic force traps the lipoproteins (247). These binding proteoglycans are produced by msSMCS which originate from cSMCs. The change occurs in the medial layer, and the msSMCs move into the deep layer of the intima. Proteoglycans are then secreted into the intima. SMCs are the first cells seen in the intima in locations destined to develop atherosclerosis (13). Prevention of the cSMC transformation would halt the binding. The chronic lipid inflammatory association would stop, as would the pathogenesis of atherosclerosis.

Vascular SMCs are subject to phenotypic change. The mechanisms resulting in transformation are complex. The plasticity of a SMC depends on its anatomic location, embryonic origin, maturity, and subtype. Most research has focused on the contractile phenotype and the synthetic phenotype. The genetic transformation involves the activation and suppression of SMC genes. PDGF can induce transformation, which is mediated by multiple mechanisms (248). One of those mechanisms was elucidated recently. A DNA-modifying enzyme ten-eleven translocation-2 (TET2) is a master epigenetic regulator of cSMC transformation (249). MicroRNA-22-3p (miR-22-3p) activates TET2 to transform cSMCs. Circular mitogen-activated protein kinase 5 (circMAP3K5) acts like a sponge for miR-22-3p. This reduction in the availability of miR-22-3p decreases the genetic transformation of cSMCs. In an *in vivo* mouse model, circMAP3K5 inhibited SMC-induced intimal hyperplasia. PDGF significantly reduces the expression of circMAP3K5 in human coronary artery SMCs (250). This reduction would lead to an increase in cSMC transformation to msSMCs. We have discussed numerous ways in which to minimize PDGF. The purpose of reducing PDGF is to reduce the creation of msSMCs.

Transformation of cSMCs into msSMCs can occur by other mechanisms. Angiopoietins are present in SMCs. They can influence genetic transformation. An increase in angiopoietin 2 (Angpt2) relative to angiopoietin 1 (Angpt1) in human aortic SMCs caused a transformation to migratory SMCs. The high-risk periodontal pathogen *Porphyromonas gingivalis* stimulated the Angpt2/Angpt1 ratio shift. The authors concluded that this mechanism links periodontal disease to atherosclerosis (95). Periodontal disease should be addressed as a CV risk factor. Protein kinase C (PKC) enhances the conversion of cSMCs to msSMCs. Insulin resistance activates PKC. Activated PKC also reduces nitric oxide and increases endothelial-1, increasing cSMC transformation (251). The solution is to minimize insulin resistance. Thromboxane A2 (TXA2) activates yes associated protein 1 (YAP)/WW-domain-containing transcription regulator 1 (TAZ), which causes more transformation of cSMCs to msSMCs (252). TXA2 is mainly synthesized *via* cyclooxygenase (COX)-1 in activated platelets. Urinary thromboxane B2 (TXB2) is a surrogate biomarker for TXA2 production. TXB2 is an independent risk factor for CV mortality. The authors suggest that TXB2 measurement could help guide the use of low-dose aspirin (253). Despite aspirin use, TXB2 levels can be elevated. Numerous health conditions can cause this. They include cigarette smoking, T2DM, HTN, obesity, and systemic lupus erythematosus. Any issue which causes OS activates platelets which increases the levels of TXA2, leading to higher TXB2 (254). To accomplish minimization of TXA2 requires managing all the CV risk factors causing OS and judicial use of low-dose aspirin.

## Translation to clinical practice

Atherosclerosis and the accompanying chronic association of lipids and inflammation is arguably the most common and devastating human ailment. Researchers have noted preventing the seeding of msSMCs into the intima as a potential solution to halt the pathogenesis of atherosclerosis (13, 255, 256). Utilization of the science presented in this paper provides a clinical path to reduce the production of msSMCs by SOS. That achievement in an individual could eliminate chronic inflammation associated with lipids. More importantly, it could establish arterial health minimizing the risk for end-stage macrovascular diseases such as myocardial infarction and ischemic stroke. Most importantly, it would minimize the risk of developing chronic microvascular diseases of aging such as dementia, heart failure, renal failure, erectile dysfunction, peripheral arterial disease, and eye conditions like macular degeneration.

Numerous health issues are known to create arterial inflammation. These conditions included physical inactivity, lipids, smoking, hypertension, poor diet, obesity, insulin resistance, periodontal disease, other chronic infections, obstructive sleep apnea, psychosocial, vitamin D deficiency, gut dysbiosis, autoimmune diseases, and air pollution. Optimal clinical management of these health conditions has been proposed to halt atherosclerosis (257). In 2015, a retrospective analysis of data from a clinic utilizing the above approach was published. The study found that such individualized care positively affected the atherosclerotic disease process. The study evaluated the change in carotid intima-media thickness (cIMT) and carotid plaque during 8 years of clinical management of 576 patients. Baseline demographics revealed a mean age of 55.5 years, mean BMI of 27.5 kg/m^2^, 39% female, 36% current or former smokers, 73% insulin resistant, 89% hyperlipidemia, 58% hypertensive, 34% Framingham Risk Score of >10% and 100% White. Carotid plaque was present in 85% of the patients. Coronary artery disease, as defined by coronary calcification or history of coronary event or intervention, was present in 25% of subjects. Eight years of therapy to improve all non-optimal health issues that created arterial inflammation resulted in a significant regression of cIMT and quantity of carotid plaque. There was also a significant reduction in Lp-PLA2, a biomarker that indicates active pathogenesis of atherosclerosis, as mentioned earlier in this paper. These results indicated not only halting arterial disease but also regressing the disease. In addition, no CV events occurred in any 576 patients (258). This information supports that knowledge regarding the causes of arterial inflammation can be translated into actual practice to stop the arterial disease.

Halting and regressing atherosclerosis theoretically should be beneficial. However, three prospective studies utilizing sophisticated imaging of arterial disease with MRI of carotids, near-infrared spectroscopy, and optical coherence tomography of the coronaries demonstrated that the lipid-richness of the plaque is the significant predictor of CV events (259–261). A retrospective study examined carotid plaque morphology changes in patients receiving multifactorial CV risk reduction treatment in the prevention clinic referenced in the above paragraph. The cohort consisted of 324 patients treated for 5 years with baseline characteristics very similar to the population of 576 patients mentioned above. At baseline, 53% of subjects had at least one lipid-rich plaque. At the end of 5 years, no patients had any lipid-rich plaque. The authors concluded that comprehensive, evidence-based management in a community-based prevention clinic benefits vulnerable plaque (262). These results indicate that clinicians can shut down and stabilize atherosclerosis with comprehensive management of health conditions causing OS and inflammation.

There are strengths in the above real-world evidence (RWE). Such evidence is increasing in importance. RWE can better mimic a patient's actual clinical situation. This reflection would include compliance with advice, number of illnesses, and magnitude of multiple treatments in an individual patient. RWE has obvious financial benefits compared to randomized clinical trials (RCT). Many unanswered questions in medicine which involve multiple variable interventions in an individual patient are not feasible to answer in RCT. RWE can also potentially generate answers faster than RCT. The expediency is especially true if the therapies are approved, making recruitment for an RCT more difficult (263). RWE is not limited to initiation by an investigator or dependent upon corporate sponsorship, which can introduce bias (264). It is now recognized that RWE is seriously needed to bolster the knowledge deficiency between RCTs and evidence-based, innovative management of complex diseases (265).

Let us examine some strengths of the RWE presented above, indicating halting and stabilizing of arterial disease. The outcome measurement of cIMT is robust. The same company performed all of the cIMT tests. Their sonographers and readers undergo routine testing for accuracy. There is a strong correlation between cIMT and atherosclerosis histology. The systemic presence of arterial disease is also correlated with cIMT. CV event risk is correlated with the change in cIMT. CV event risk decreases 12% for each 0.010 mm yearly reduction in cIMT progression (266). The RWE showed regression of cIMT instead of simply slowing progression. Identifying subclinical atherosclerosis with carotid plaque was clinically valuable in determining comprehensiveness and management goals. The presence of atherosclerosis is *conditio sine qua non* for risk of having a CV event (267). Finding atheroma in “healthy” subjects enhances patient management decisions (268). Subclinical carotid plaque is strongly associated with heart attack and stroke risk. Such identification should reclassify an individual's risk for a CV event (269). Identifying subclinical arterial disease is a cornerstone of the prevention clinic. This investigation allowed the migration to a ternary classification of CV prevention. Primary prevention care was provided to patients without any arterial disease. Secondary prevention was delivered to individuals with subclinical arterial disease. Tertiary prevention was given to subjects who proved they had arterial disease with interventions or events. This classification aided therapy decisions such as low-dose aspirin (270). The high degree of management compliance is partially attributed to the patient's visualization of the plaque (271). The cIMT testing enhanced care, and the follow-up results proved that atherosclerosis's pathogenesis could be halted in an actual healthcare clinic.

Another strength of the RWE involves the monitoring of arterial inflammation. The prevention clinic assessed the adequacy of management of all the causes of arterial inflammation with these biomarkers. The endothelium was judged for the amount of inflammation by the hsCRP and wellness by the microalbumin-creatinine ratio (272, 273). Intimal inflammation and atherogenic activity were checked with lipoprotein-associated phospholipase A2 (Lp-PLA2) (189). Myeloperoxidase (MPO) was monitored since elevations are associated with an increased risk of endothelial rupture and erosion (274). Urinary F2-isoprostane was followed as a marker of oxidative stress (275). Clinically, the patient was judged to have a quiescent atherosclerotic process and low risk for an atherothrombotic event when all the inflammatory biomarkers were in an acceptable range. This approach was successful, albeit without a complete understanding of the reasons. As explained throughout this paper, we know that all the root causes of inflammation being managed in the clinic generate OS, leading to lipoprotein retention in the intima. The trapped lipoproteins then turn on the immune system, which causes the monitored biomarkers of inflammation to increase. With mitigation of OS, the first step in atherosclerosis's pathogenesis is prevented, ending the chronic association of lipids and inflammation. Once the atherosclerotic disease process stops, any existing atheroma has an opportunity to delipidate and shrink. The RWE showing a significant reduction in Lp-PLA2 supports this concept.

One glaring weakness of the RWE is that there was a lack of ethnic diversity. The good news is that from a biological standpoint, the principle of SOS should halt and stabilize arterial disease regardless of ethnicity. Humans are 99.99% identical from a genetic standpoint. There are different frequencies of genetic variants in ethnic groups, but humans are arguably the same race. Discrete human ethnic groups are based on social constructs such as language, neighborhoods, education, income, preferred foods, and physical activity (276). Biological responses to these social differences will affect the degree of OS. Therefore, any study evaluating the RWE in other ethnic populations must accurately assess the environmental and sociocultural issues affecting the risk factors that generate OS (277). Given this, arterial disease can be halted and stabilized in humans.

The RWE presented supports that the chronic association of lipids and inflammation can be interrupted. SOS can halt the pathogenesis of atherosclerosis. Clinically this requires individual patient management of the myriad of conditions that cause OS. Such management necessitates a time-consuming individualized interdisciplinary healthcare approach. An excellent term for such care is arteriology. There is a vast need for arteriologists. The RWE should be bolstered in additional clinics and populations. It would be challenging to design RCTs to strengthen the data due to the need for personalized care manipulating numerous variations in management. The current RWE is logical and anchored in evidence-based therapies. There is a new meaning for the distress code- SOS, which could enhance the lives of millions.

For interested individuals, the prevention clinic with the RWE has a nomenclature for the system of care delivered. It is called the BaleDoneen Method. Instruction in the method is delivered to other healthcare providers in an approved 17-h CME and CE course. The public can learn about the method from two books [Bale, Bradley F., Doneen, Amy L., & Cool, Lisa Collier (2014). Beat the Heart Attack Gene: The Revolutionary Plan to Prevent Heart Disease, Stroke, and Diabetes. Nashville, TN: John Wiley & Sons.; Bale, Bradley F., Doneen, Amy L., & Cool, Lisa Collier (2022). Healthy Heart Healthy Brain: The Personalized Path to Protect Your Memory, Prevent Heart Attacks and Strokes and Avoid Chronic Illness. New York, NY: Little, Brown Spark].

## Author contributions

All authors listed have made a substantial, direct, and intellectual contribution to the work and approved it for publication.

## Conflict of interest

The authors declare that the research was conducted in the absence of any commercial or financial relationships that could be construed as a potential conflict of interest.

## Publisher's note

All claims expressed in this article are solely those of the authors and do not necessarily represent those of their affiliated organizations, or those of the publisher, the editors and the reviewers. Any product that may be evaluated in this article, or claim that may be made by its manufacturer, is not guaranteed or endorsed by the publisher.

## Abbreviations

ACL: ATP citrate lyase
ADMA: Asymmetrical dimethylarginine
AHI: Apneas or hypopneas per hour
AMPK: Adenosine monophosphate-activated protein kinase
AngII: Angiotensin II
Angpt1: Angiopoietin−1
Angpt2: Angiopoietin 2
apoB: Apolipoprotein B
AQG: Air quality guidelines
ATP: Adenosine triphosphate
BA: Bempedoic acid
BB: Beta blockers
BMI: Body mass index
CAD: Coronary artery disease
CAT: Catalase
CCB: Calcium channel blockers
CKD: Chronic kidney disease
cIMT: Carotid intima-medial thickness
circMAP3K5: Circular mitogen-activated protein kinase−5
COX-2: Cyclooxygenase−2
CPAP: Continuous positive airway pressure
cSMC: Contractile smooth muscle cells
CV: Cardiovascular
CVD: Cardiovascular disease
EC-SOD: Extracellular superoxide dismutase
ED: Endothelial dysfunction
eNOS: endothelial nitric oxide synthase
ET-1: Endothelin-1
FA: Fatty acids
FRAP: Ferric-reducing ability of plasma
GGPP: geranyl-geranyl-pyrophosphate
GLP-1: Glucagon-like peptide-1
GLUT4: Glucose transporter−4
GPx: Glutathione peroxidase
GSH: Glutathione
HCV: Hepatitis C virus
HIV: Human immunodeficiency virus
HMG-CoA: 3-hydroxy-3-methylglutaryl coenzyme A
HTN: Hypertension
IDL: Intermediate-density lipoprotein
IR: Insulin resistance
LDL: Low-density lipoprotein
LDL-C: Low-density-lipoprotein cholesterol
MDA: Malondialdehyde
miR-22-3: MicroRNA 22-3
MPO: Myeloperoxidase
MRI: Magnetic resonance imaging
msSMC: Migratory secretory smooth muscle cells
mtDNA: Mitochondrial Deoxynucleic acid
NADPH: Nicotinamide adenine dinucleotide phosphate
NF-κB: Nuclear factor kappa B
NLRP3: (NOD)-like receptor protein-3
NO: Nitric oxide
Nrf2: Nuclear factor erythroid 2-related factor 2
OA: Oral appliance
OS: Oxidative stress
OSA: Obstructive sleep apnea
PCSK-9: Proprotein convertase subtilisin/kexin type 9
PDGF: Platelet-derived growth factor
PKC: Protein Kinase C
PLAC2: Lipoprotein-associated phospholipase A-2
RA: Rheumatoid arthritis
RAAS: Renin-angiotensin-aldosterone system
RCT: Randomized clinical trials
RNS: Reactive nitrogen species
ROS: Reactive oxygen species
RWE: Real world evidence
SGLT-2: Sodium-glucose cotransporter 2
SIRT3: Mitochondrial deacetylase sirtuin 3
SLE: Systemic lupus erythematosus
SOD: Superoxide dismutase
SOS: Stopping oxidative stress
T2DM: Type II diabetes Meletus
TBARS: Thiobarbituric acid-reactive substances
TET2: ten-eleven translocation-2
TG: Triglyceride
TGFβ: Tumor growth factor β
TLR: Toll-like receptors
TNFα: Tumor necrosis factor α
TXA2: Thromboxane A2
TXB2: Thromboxane B2
TZD: Thiazolidinediones
VLDL: Very-low-density lipoprotein.

## References

[1] Tsao CW Aday AW Almarzooq ZI Alonso A Beaton AZ Bittencourt MS. Heart disease and stroke statistics-2022 update: a report from the American Heart Association. Circulation. (2022) 145:e153–639. 10.1161/CIR.0000000000001052 35078371

[2] Hakim AM. Small vessel disease. Front Neurol. (2019) 10:1020. 10.3389/fneur.2019.01020 31616367 PMC6768982

[3] Virani SS Alonso A Benjamin EJ Bittencourt MS Callaway CW Carson AP. Heart disease and stroke statistics-2020 update: a report from the American Heart Association. Circulation. (2020) 141:e139–596. 10.1161/CIR.0000000000000757 31992061

[4] Schwartz RS Burke A Farb A Kaye D Lesser JR Henry TD. Microemboli and microvascular obstruction in acute coronary thrombosis and sudden coronary death: relation to epicardial plaque histopathology. J Am Coll Cardiol. (2009) 54:2167–73. 10.1016/j.jacc.2009.07.042 19942088

[5] Bezsonov E Sukhorukov V Bukrinsky M Orekhov A. Editorial: lipids and inflammation in health and disease. Front Cardiovasc Med. (2022) 9:864429. 10.3389/fcvm.2022.864429 35369350 PMC8964976

[6] Nakashima Y Fujii H Sumiyoshi S Wight TN Sueishi K. Early human atherosclerosis: accumulation of lipid and proteoglycans in intimal thickenings followed by macrophage infiltration. Arterioscler Thromb Vasc Biol. (2007) 27:1159–65. 10.1161/ATVBAHA.106.134080 17303781

[7] Fogelstrand P Borén J. Retention of atherogenic lipoproteins in the artery wall and its role in atherogenesis. Nutr Metab Cardiovasc Dis. (2012) 22:1–7. 10.1016/j.numecd.2011.09.007 22176921

[8] Davignon J Ganz P. Role of endothelial dysfunction in atherosclerosis. Circulation. (2004) 109:III-27–32. 10.1161/01.CIR.0000131515.03336.f8 15198963

[9] He D Xu L Wu Y Yuan Y Wang Y Liu Z. Rac3, but not Rac1, promotes ox-LDL induced endothelial dysfunction by downregulating autophagy. J Cell Physiol. (2020) 235:1531–42. 10.1002/jcp.29072 31332791

[10] Li D Mehta JL. Antisense to LOX-1 inhibits oxidized LDL–mediated upregulation of monocyte chemoattractant protein-1 and monocyte adhesion to human coronary artery endothelial cells. Circulation. (2000) 101:2889–95. 10.1161/01.CIR.101.25.2889 10869259

[11] Hua Y Zhang J Liu Q Su J Zhao Y Zheng G. The induction of endothelial autophagy and its role in the development of atherosclerosis. Front Cardiovasc Med. (2022) 9:831847. 10.3389/fcvm.2022.831847 35402552 PMC8983858

[12] Nakashima Y Wight TN Sueishi K. Early atherosclerosis in humans: role of diffuse intimal thickening and extracellular matrix proteoglycans. Cardiovasc Res. (2008) 79:14–23. 10.1093/cvr/cvn099 18430750

[13] Doran AC Meller N McNamara CA. Role of smooth muscle cells in the initiation and early progression of atherosclerosis. Arteriosc Thromb Vasc Biol. (2008) 28:812–9. 10.1161/ATVBAHA.107.159327 18276911 PMC2734458

[14] Ricci C Ferri N. Naturally occurring PDGF receptor inhibitors with potential anti-atherosclerotic properties. Vascul Pharmacol. (2015) 70:1–7. 10.1016/j.vph.2015.02.002 25737405

[15] Freedman JE. Oxidative stress and platelets. Arteriosc Thromb Vasc Biol. (2008) 28:s11–6. 10.1161/ATVBAHA.107.159178 18174453

[16] Rossi E Casali B Regolisti G Davoli S Perazzoli F Negro A. Increased plasma levels of platelet-derived growth factor (PDGF-BB + PDGF-AB) in patients with never-treated mild essential hypertension. Am J Hypertens. (1998) 11:1239–43. 10.1016/S0895-7061(98)00124-1 9799041

[17] Lichtenberg D Pinchuk I. Oxidative stress, the term and the concept. Biochem Biophys Res Commun. (2015) 461:441–4. 10.1016/j.bbrc.2015.04.062 25911322

[18] Tirichen H Yaigoub H Xu W Wu C Li R Li Y. Mitochondrial reactive oxygen species and their contribution in chronic kidney disease progression through oxidative stress. Front Physiol. (2021) 12:627837. 10.3389/fphys.2021.627837 33967820 PMC8103168

[19] Mailloux RJ. An update on mitochondrial reactive oxygen species production. Antioxidants. (2020) 9:472. 10.3390/antiox9060472 32498250 PMC7346187

[20] JeŽek J Engstová H JeŽek P. Antioxidant mechanism of mitochondria-targeted plastoquinone SkQ1 is suppressed in aglycemic HepG2 cells dependent on oxidative phosphorylation. Biochimica et Biophysica Acta Bioenergetics. (2017) 1858:750–762. 10.1016/j.bbabio.2017.05.005 28554565

[21] Leyane TS Jere SW Houreld NN. Oxidative stress in ageing and chronic degenerative pathologies: molecular mechanisms involved in counteracting oxidative stress and chronic inflammation. Int J Mol Sci. (2022) 23:7273. 10.3390/ijms23137273 35806275 PMC9266760

[22] Jiang Q Yin J Chen J Ma X Wu M Liu G. Mitochondria-targeted antioxidants: a step towards disease treatment. Oxid Med Cell Longev. (2020) 2020:1–18. 10.1155/2020/8837893 33354280 PMC7735836

[23] Shemiakova T Ivanova E Wu WK Kirichenko TV Starodubova AV Orekhov AN. Atherosclerosis as mitochondriopathy: repositioning the disease to help finding new therapies. Front Cardiovasc Med. (2021) 8:660473. 10.3389/fcvm.2021.660473 34017868 PMC8129197

[24] Zhang M-J Zhou Y Chen L Wang Y-Q Wang X Pi Y. An overview of potential molecular mechanisms involved in VSMC phenotypic modulation. Histochem Cell Biol. (2016) 145:119–30. 10.1007/s00418-015-1386-3 26708152

[25] Sniderman AD Navar AM Thanassoulis G. Apolipoprotein B vs low-density lipoprotein cholesterol and non–high-density lipoprotein cholesterol as the primary measure of apolipoprotein B lipoprotein-related risk. JAMA Cardiology. (2022) 7:257. 10.1001/jamacardio.2021.5080 34773457

[26] Varbo A Benn M Tybjaerg-Hansen A Nordestgaard BG. Elevated remnant cholesterol causes both low-grade inflammation and ischemic heart disease, while elevated low-density lipoprotein cholesterol causes ischemic heart disease without inflammation. Circulation. (2013) 128:1298–309. 10.1161/CIRCULATIONAHA.113.003008 23926208

[27] Le NA. Lipoprotein-associated oxidative stress: a new twist to the postprandial hypothesis. Int J Mol Sci. (2014) 16:401–19. 10.3390/ijms16010401 25548897 PMC4307253

[28] Paolisso G Gambardella A Tagliamonte MR Saccomanno F Salvatore T Gualdiero P. Does free fatty acid infusion impair insulin action also through an increase in oxidative stress? J Clin Endocrinol Metab. (1996) 81:4244–8. 10.1210/jcem.81.12.8954022 8954022

[29] Soardo G Donnini D Domenis L Catena C De Silvestri D Cappello D. Oxidative stress is activated by free fatty acids in cultured human hepatocytes. Metab Syndr Relat Disord. (2011) 9:397–401. 10.1089/met.2010.0140 21561340

[30] Shin HK Kim YK Kim KY Lee JH Hong KW. Remnant lipoprotein particles induce apoptosis in endothelial cells by NAD(P)H oxidase-mediated production of superoxide and cytokines via lectin-like oxidized low-density lipoprotein receptor-1 activation: prevention by cilostazol. Circulation. (2004) 109:1022–8. 10.1161/01.CIR.0000117403.64398.53 14967724

[31] Castañer O Pintó X Subirana I Amor AJ Ros E Hernáez Á. Remnant cholesterol, not LDL cholesterol, is associated with incident cardiovascular disease. J Am Coll Cardiol. (2020) 76:2712–24. 10.1016/j.jacc.2020.10.008 33272365

[32] Sascău R Clement A Radu R Prisacariu C Stătescu C. Triglyceride-rich lipoproteins and their remnants as silent promoters of atherosclerotic cardiovascular disease and other metabolic disorders: a review. Nutrients. (2021) 13:1774. 10.3390/nu13061774 34067469 PMC8224751

[33] Wadström BN Wulff AB Pedersen KM Jensen GB Nordestgaard BG. Elevated remnant cholesterol increases the risk of peripheral artery disease, myocardial infarction, and ischaemic stroke: a cohort-based study. Eur Heart J. (2021) 43:3258–3269. 10.1093/eurheartj/ehab705 34661640

[34] Zhao Y Liu L Yang S Liu G Pan L Gu C. Mechanisms of atherosclerosis induced by postprandial lipemia. Front Cardiovasc Med. (2021) 8:636947. 10.3389/fcvm.2021.636947 33996937 PMC8116525

[35] Wang JL Yin WJ Zhou LY Wang YF Zuo XC. Association between initiation, intensity, and cessation of smoking and mortality risk in patients with cardiovascular disease: a cohort study. Front Cardiovasc Med. (2021) 8:728217. 10.3389/fcvm.2021.728217 34977166 PMC8714779

[36] Hernandez P Passi N Modarressi T Kulkarni V Soni M Burke F. Clinical management of hypertriglyceridemia in the prevention of cardiovascular disease and pancreatitis. Curr Atherosc Rep. (2021) 23:72. 10.1007/s11883-021-00962-z 34515873 PMC8436578

[37] Fu J Bonder MJ Cenit MC Tigchelaar EF Maatman A Dekens JAM. The gut microbiome contributes to a substantial proportion of the variation in blood lipids. Circ Res. (2015) 117:817–24. 10.1161/CIRCRESAHA.115.306807 26358192 PMC4596485

[38] O'Donnell MJ Xavier D Liu L Zhang H Chin SL Rao-Melacini P. Risk factors for ischaemic and intracerebral haemorrhagic stroke in 22 countries (the INTERSTROKE study): a case-control study. Lancet. (2010) 376:112–23. 10.1016/S0140-6736(10)60834-3 20561675

[39] Touyz RM Rios FJ Alves-Lopes R Neves KB Camargo LL Montezano AC. Oxidative stress: a unifying paradigm in hypertension. Canadian J Cardiol. (2020) 36:659–70. 10.1016/j.cjca.2020.02.081 32389339 PMC7225748

[40] Li FJ Zhang CL Luo XJ Peng J Yang TL. Involvement of the MiR-181b-5p/HMGB1 pathway in ang ii-induced phenotypic transformation of smooth muscle cells in hypertension. Aging Dis. (2019) 10:231–48. 10.14336/AD.2018.0510 31011475 PMC6457049

[41] Wright JT Jr Williamson JD Whelton PK Snyder JK Sink KM Rocco MV. A randomized trial of intensive versus standard blood-pressure control. N Engl J Med. (2015) 373:2103–16. 10.1056/NEJMoa1511939 26551272 PMC4689591

[42] Zhang W Zhang S Deng Y Wu S Ren J Sun G. Trial of intensive blood-pressure control in older patients with hypertension. New Engl J Med. (2021) 385:1268–79. 10.1056/NEJMoa2111437 34491661

[43] Verma N Rastogi S Chia YC Siddique S Turana Y Cheng HM. Non-pharmacological management of hypertension. J Clin Hypertens. (2021) 23:1275–83. 10.1111/jch.14236 33738923 PMC8678745

[44] Jones NR McCormack T Constanti M McManus RJ. Diagnosis and management of hypertension in adults: NICE guideline update 2019. Br J General Pract. (2020) 70:90–1. 10.3399/bjgp20X708053 32001477 PMC7018407

[45] Noubiap JJ Nansseu JR Nyaga UF Sime PS Francis I Bigna JJ. Global prevalence of resistant hypertension: a meta-analysis of data from 3.2 million patients. Heart. (2019) 105:98–105. 10.1136/heartjnl-2018-313599 30087099

[46] Yoshiyama S Chen Z Okagaki T Kohama K Nasu-Kawaharada R Izumi T. Nicotine exposure alters human vascular smooth muscle cell phenotype from a contractile to a synthetic type. Atherosclerosis. (2014) 237:464–70. 10.1016/j.atherosclerosis.2014.10.019 25463075

[47] Schroeder SA. What to do with a patient who smokes. JAMA. (2005) 294:482–7. 10.1001/jama.294.4.482 16046655

[48] Brewer NT Hall MG Noar SM Parada H Stein-Seroussi A Bach LE. Effect of pictorial cigarette pack warnings on changes in smoking behavior: a randomized clinical trial. JAMA Intern Med. (2016) 176:905–12. 10.1001/jamainternmed.2016.2621 27273839 PMC5458743

[49] Martins RS Junaid MU Khan MS Aziz N Fazal ZZ Umoodi M. Factors motivating smoking cessation: a cross-sectional study in a lower-middle-income country. BMC Public Health. (2021) 21:1419. 10.1186/s12889-021-11477-2 34275456 PMC8286564

[50] Vinci C Lam C Schlechter CR Shono Y Vidrine JI Wetter DW. Increasing treatment enrollment among smokers who are not motivated to quit: a randomized clinical trial. Transl Behav Med. (2022) 12: ibab114. 10.1093/tbm/ibab114 34424337 PMC8764989

[51] Haffner SM Lehto S Rönnemaa T Pyörälä K Laakso M. Mortality from coronary heart disease in subjects with type 2 diabetes and in nondiabetic subjects with and without prior myocardial infarction. New Engl J Med. (1998) 339:229–34. 10.1056/NEJM199807233390404 9673301

[52] Goldstein BJ. Insulin resistance as the core defect in type 2 diabetes mellitus. Am J Cardiol. (2002) 90:3–10. 10.1016/S0002-9149(02)02553-5 12231073

[53] World Health Organization. Global Report on Diabetes. Geneva: World Health Organization (2016).

[54] Centers for Disease Control and Prevention. National Diabetes Statistics Report: Estimates of Diabetes and Its Burden in the United States, 2014. Atlanta, GA: US Department of Health and Human Services (2014).

[55] Yu Z Cheng J-X Zhang D Yi F Ji Q. Association between obstructive sleep apnea and type 2 diabetes mellitus: a dose-response meta-analysis. Evid Based Complement Alternat Med. (2021) 2021:1–14. 10.1155/2021/1337118 34630603 PMC8497107

[56] Choi HS Kim K-A Lim C-Y Rhee SY Hwang Y-C Kim KM. Low serum vitamin D is associated with high risk of diabetes in korean adults. J Nutr. (2011) 141:1524–8. 10.3945/jn.111.139121 21697301

[57] Brauchli YB Jick SS Meier CR. Psoriasis and the risk of incident diabetes mellitus: a population-based study. Br J Dermatol. (2008) 159:1331–7. 10.1111/j.1365-2133.2008.08814.x 18782318

[58] Holmlund A Lind L. Periodontal disease and a poor response to periodontal treatment were associated with an increased risk of incident diabetes: a longitudinal cohort study in Sweden. J Clin Periodontol. (2021) 48:1605–12. 10.1111/jcpe.13558 34605049

[59] Zhao J Zhang Y Wei F Song J Cao Z Chen C. Triglyceride is an independent predictor of type 2 diabetes among middle-aged and older adults: a prospective study with 8-year follow-ups in two cohorts. J Transl Med. (2019) 17:403. 10.1186/s12967-019-02156-3 31801571 PMC6894231

[60] Tsimihodimos V Gonzalez-Villalpando C Meigs JB Ferrannini E. Hypertension and diabetes mellitus. Hypertension. (2018) 71:422–28. 10.1161/HYPERTENSIONAHA.117.10546 29335249 PMC5877818

[61] Harris ML Oldmeadow C Hure A Luu J Loxton D Attia J. Stress increases the risk of type 2 diabetes onset in women: a 12-year longitudinal study using causal modelling. PLoS ONE. (2017) 12:e0172126. 10.1371/journal.pone.0172126 28222165 PMC5319684

[62] Jeong D Karim ME Wong S Wilton J Butt ZA Binka M. Impact of HCV infection and ethnicity on incident type 2 diabetes: findings from a large population-based cohort in British Columbia. BMJ Open Diabetes Res Care. (2021) 9:e002145. 10.1136/bmjdrc-2021-002145 34099439 PMC8186745

[63] Chen H Burnett RT Kwong JC Villeneuve PJ Goldberg MS Brook RD. Risk of incident diabetes in relation to long-term exposure to fine particulate matter in Ontario, Canada. Environ Health Perspect. (2013) 121:804–810. 10.1289/ehp.1205958 23632126 PMC3701997

[64] Atuegwu NC Perez MF Oncken C Mead EL Maheshwari N Mortensen EM. E-cigarette use is associated with a self-reported diagnosis of prediabetes in never cigarette smokers: results from the behavioral risk factor surveillance system survey. Drug Alcohol Depend. (2019) 205:107692. 10.1016/j.drugalcdep.2019.107692 31707269 PMC6893144

[65] Luca M Di Mauro M Di Mauro M Luca A. Gut microbiota in Alzheimer's disease, depression, and type 2 diabetes mellitus: the role of oxidative stress. Oxid Med Cell Longev. (2019) 2019:1–10. 10.1155/2019/4730539 31178961 PMC6501164

[66] Heinecke JW Goldberg IJ. Myeloperoxidase: a therapeutic target for preventing insulin resistance and the metabolic sequelae of obesity? Diabetes. (2014) 63:4001–3. 10.2337/db14-1273 25414015 PMC4238000

[67] Bondonno NP Davey RJ Murray K Radavelli-Bagatini S Bondonno CP Blekkenhorst LC. Associations between fruit intake and risk of diabetes in the AusDiab cohort. J Clin Endocrinol Metab. (2021) 106:e4097–108. 10.1210/clinem/dgab335 34076673 PMC8475213

[68] Sawada SS Lee I-M Naito H Noguchi J Tsukamoto K Muto T. Long-term trends in cardiorespiratory fitness and the incidence of type 2 diabetes. Diabetes Care. (2010) 33:1353–7. 10.2337/dc09-1654 20215460 PMC2875453

[69] Hurrle S Hsu WH. The etiology of oxidative stress in insulin resistance. Biomed J. (2017) 40:257–62. 10.1016/j.bj.2017.06.007 29179880 PMC6138814

[70] Defronzo RA. Banting Lecture. From the triumvirate to the ominous octet: a new paradigm for the treatment of type 2 diabetes mellitus. Diabetes. (2009) 58:773–95. 10.2337/db09-9028 19336687 PMC2661582

[71] Shan Z Li Y Baden MY Bhupathiraju SN Wang DD Sun Q. Association between healthy eating patterns and risk of cardiovascular disease. JAMA Intern Med. (2020) 10.1001/jamainternmed.2020.2176 32539102 PMC7296454

[72] Venkatesh R Sood D. A Review of the Physiological Implications of Antioxidants in Food. Bachelor of Science Interactive Qualifying Project. Worcester, Massachusetts, US: Worcester Polytechnic Institute (2011).

[73] Redman LM Smith SR Burton JH Martin CK Il'Yasova D Ravussin E. Metabolic slowing and reduced oxidative damage with sustained caloric restriction support the rate of living and oxidative damage theories of aging. Cell Metab. (2018) 27:805–15.e4. 10.1016/j.cmet.2018.02.019 29576535 PMC5886711

[74] Gabel K Cienfuegos S Kalam F Ezpeleta M Varady KA. Time-restricted eating to improve cardiovascular health. Curr Atheroscler Rep. (2021) 23:22. 10.1007/s11883-021-00922-7 33772388 PMC8218778

[75] Pérez-Beltrán YE Rivera-Iñiguez I Gonzalez-Becerra K Pérez-Naitoh N Tovar J Sáyago-Ayerdi SG. Personalized dietary recommendations based on lipid-related genetic variants: a systematic review. Front Nutr. (2022) 9:830283. 10.3389/fnut.2022.830283 35387194 PMC8979208

[76] Hooker SP Diaz KM Blair SN Colabianchi N Hutto B McDonnell MN. Association of accelerometer-measured sedentary time and physical activity with risk of stroke among US adults. JAMA Network Open. (2022) 5:e2215385. 10.1001/jamanetworkopen.2022.15385 35657625 PMC9166254

[77] Derbré F Gratas-Delamarche A Gómez-Cabrera MC Viña J. Inactivity-induced oxidative stress: a central role in age-related sarcopenia? Eur J Sport Sci. (2014) 14:S98–108. 10.1080/17461391.2011.654268 24444251

[78] Kozakova M Palombo C. Vascular ageing and aerobic exercise. Int J Environ Res Public Health. (2021) 18:10666. 10.3390/ijerph182010666 34682413 PMC8535583

[79] Hogstrom G Nordstrom A Nordstrom P. High aerobic fitness in late adolescence is associated with a reduced risk of myocardial infarction later in life: a nationwide cohort study in men. Eur Heart J. (2014) 35:3133–40. 10.1093/eurheartj/eht527 24398666

[80] Fabris E Sinagra G. Physical activity in older people: better late than never, but better early than late. Heart. (2022) 108:328–9. 10.1136/heartjnl-2021-320462 35165167

[81] Du Y Liu B Sun Y Snetselaar LG Wallace RB Bao W. Trends in adherence to the physical activity guidelines for americans for aerobic activity and time spent on sedentary behavior among US adults, 2007 to 2016. JAMA Network Open. (2019) 2:e197597. 10.1001/jamanetworkopen.2019.7597 31348504 PMC6661709

[82] Ferrari G Cristi-Montero C Drenowatz C Kovalskys I Gómez G Rigotti A. Meeting 24-h movement guidelines and markers of adiposity in adults from eight Latin America countries: the ELANS study. Sci Rep. (2022) 12:11382. 10.1038/s41598-022-15504-z 35790777 PMC9256603

[83] Lloyd-Jones DM Allen NB Anderson CAM Black T Brewer LC Foraker RE. Life's essential 8: updating and enhancing the American Heart Association's construct of cardiovascular health: a presidential advisory from the American Heart Association. Circulation. (2022) 146:e18–43. 10.1161/CIR.0000000000001078 35766027 PMC10503546

[84] Khan SS Ning H Wilkins JT Allen N Carnethon M Berry JD. Association of body mass index with lifetime risk of cardiovascular disease and compression of morbidity. JAMA Cardiol. (2018) 3:280–7. 10.1001/jamacardio.2018.0022 29490333 PMC5875319

[85] Zhou Z Macpherson J Gray SR Gill JMR Welsh P Celis-Morales C. Are people with metabolically healthy obesity really healthy? A prospective cohort study of 381,363 UK Biobank participants. Diabetologia. (2021) 64:1963–72. 10.1007/s00125-021-05484-6 34109441 PMC8382657

[86] Savini I Catani MV Evangelista D Gasperi V Avigliano L. Obesity-associated oxidative stress: strategies finalized to improve redox state. Int J Mol Sci. (2013) 14:10497–538 10.3390/ijms140510497 23698776 PMC3676851

[87] Galindo Muñoz JS Morillas-Ruiz JM Gómez Gallego M Díaz Soler I Barberá Ortega MDC Martínez CM. Cognitive training therapy improves the effect of hypocaloric treatment on subjects with overweight/obesity: a randomised clinical trial. Nutrients. (2019) 11:925. 10.3390/nu11040925 31022980 PMC6521325

[88] Salminen P Grönroos S Helmiö M Hurme S Juuti A Juusela R. Effect of laparoscopic sleeve gastrectomy vs Roux-en-Y gastric bypass on weight loss, comorbidities, and reflux at 10 years in adult patients with obesity. JAMA Surg. (2022) 157:656–66. 10.1001/jamasurg.2022.2229 35731535 PMC9218929

[89] Gagliardi AC Miname MH Santos RD. Uric acid: a marker of increased cardiovascular risk. Atherosclerosis. (2009) 202:11–7. 10.1016/j.atherosclerosis.2008.05.022 18585721

[90] Gaubert M Bardin T Cohen-Solal A Diévart F Fauvel JP Guieu R. Hyperuricemia and hypertension, coronary artery disease, kidney disease: from concept to practice. Int J Mol Sci. (2020) 21:4066. 10.3390/ijms21114066 33561034 PMC7312288

[91] Liu N Xu H Sun Q Yu X Chen W Wei H. The role of oxidative stress in hyperuricemia and xanthine oxidoreductase (XOR) inhibitors. Oxid Med Cell Longev. (2021) 2021:1–15. 10.1155/2021/1470380 33854690 PMC8019370

[92] Monroy MA. Chronic kidney disease alters vascular smooth muscle cell phenotype. Front Biosci. (2015) 20:784–95. 10.2741/4337 25553479 PMC4331020

[93] Gianos E Jackson EA Tejpal A Aspry K O'Keefe J Aggarwal M. Oral health and atherosclerotic cardiovascular disease: a review. Am J Prev Cardiol. (2021) 7:100179. 10.1016/j.ajpc.2021.100179 34611631 PMC8387275

[94] Bale BF Doneen AL Vigerust DJ. High-risk periodontal pathogens contribute to the pathogenesis of atherosclerosis. Postgrad Med J. (2016) 93:215–20. 10.1136/postgradmedj-2016-134279 27899684 PMC5520251

[95] Zhang B Khalaf H Sirsjo A Bengtsson T. Gingipains from the periodontal pathogen porphyromonas gingivalis play a significant role in regulation of angiopoietin 1 and angiopoietin 2 in human aortic smooth muscle cells. Infect Immun. (2015) 83:4256–65. 10.1128/IAI.00498-15 26283334 PMC4598411

[96] Sharma P Fenton A Dias IH Heaton B Brown CL Sidhu A. Oxidative stress links periodontal inflammation and renal function. J Clin Periodontol. (2021) 48:357–67. 10.1111/jcpe.13414 33368493 PMC7986430

[97] da Silva JC Muniz WMG F Oballe HJR Andrades M Rösing CK Cavagni J. The effect of periodontal therapy on oxidative stress biomarkers: a systematic review. J Clin Periodontol. (2018) 45:1222–37. 10.1111/jcpe.12993 30076616

[98] Eke PI Dye BA Wei L Slade GD Thornton-Evans GO Borgnakke WS. Update on prevalence of periodontitis in adults in the United States: NHANES 2009 to 2012. J Periodontol. (2015) 86:611–22. 10.1902/jop.2015.140520 25688694 PMC4460825

[99] Eke PI Wei L Borgnakke WS Thornton-Evans G Zhang X Lu H. Periodontitis prevalence in adults ≥ 65 years of age, in the USA. Periodontol 2000. (2016) 72:76–95. 10.1111/prd.12145 27501492 PMC8223257

[100] Kassebaum NJ Bernabé E Dahiya M Bhandari B Murray CJ Marcenes W. Global burden of severe periodontitis in 1990-2010: a systematic review and meta-regression. J Dent Res. (2014) 93:1045–53. 10.1177/0022034514552491 25261053 PMC4293771

[101] Domont F Cacoub P. Chronic hepatitis C virus infection, a new cardiovascular risk factor? Liver International. (2016) 36:621–7. 10.1111/liv.13064 26763484

[102] Farrugia PM Lucariello R Coppola JT. Human immunodeficiency virus and atherosclerosis. Cardiol Rev. (2009) 17:211–5. 10.1097/CRD.0b013e3181b151a3 19690471

[103] Ivanov AV Bartosch B Isaguliants MG. Oxidative stress in infection and consequent disease. Oxid Med Cell Longev. (2017) 2017:3496043. 10.1155/2017/3496043 28255425 PMC5309413

[104] Roguljic H Nincevic V Bojanic K Kuna L Smolic R Vcev A. Impact of DAA treatment on cardiovascular disease risk in chronic HCV infection: an update. Front Pharmacol. (2021) 12:678546. 10.3389/fphar.2021.678546 34045969 PMC8144519

[105] Domingues EAM Ferrit-Martín M Calleja-Hernández MÁ. Impact of pharmaceutical care on cardiovascular risk among older HIV patients on antiretroviral therapy. Int J Clin Pharm. (2017) 39:52–60. 10.1007/s11096-016-0387-1 27864732

[106] Loke YK Brown JW Kwok CS Niruban A Myint PK. Association of obstructive sleep apnea with risk of serious cardiovascular events: a systematic review and meta-analysis. Circ Cardiovasc Qual Outcomes. (2012) 5:720–8. 10.1161/CIRCOUTCOMES.111.964783 22828826

[107] Drager LF Togeiro SM Polotsky VY Lorenzi-Filho G. Obstructive sleep apnea: a cardiometabolic risk in obesity and the metabolic syndrome. J Am Coll Cardiol. (2013) 62:569–76. 10.1016/j.jacc.2013.05.045 23770180 PMC4461232

[108] Salman LA Shulman R Cohen JB. Obstructive sleep apnea, hypertension, and cardiovascular risk: epidemiology, pathophysiology, and management. Curr Cardiol Rep. (2020) 22:6. 10.1007/s11886-020-1257-y 31955254

[109] Linz D McEvoy RD Cowie MR Somers VK Nattel S Levy P. Associations of obstructive sleep apnea with atrial fibrillation and continuous positive airway pressure treatment: a review. JAMA Cardiol. (2018) 3:532–40. 10.1001/jamacardio.2018.0095 29541763

[110] Khattak HK Hayat F Pamboukian SV Hahn HS Schwartz BP Stein PK. Obstructive sleep apnea in heart failure: review of prevalence, treatment with continuous positive airway pressure, and prognosis. Tex Heart Inst J. (2018) 45:151–61. 10.14503/THIJ-15-5678 30072851 PMC6059510

[111] Arnaud C Bochaton T Pepin JL Belaidi E. Obstructive sleep apnoea and cardiovascular consequences: pathophysiological mechanisms. Arch Cardiovasc Dis. (2020) 113:350–8. 10.1016/j.acvd.2020.01.003 32224049

[112] Punjabi NM. The epidemiology of adult obstructive sleep apnea. Proc Am Thorac Soc. (2008) 5:136–43. 10.1513/pats.200709-155MG 18250205 PMC2645248

[113] American Psychiatric Association. Diagnostic Statistical Manual of Mental Disorders: DSM-5-TR. Washington, DC: American Psychiatric Association Publishing (2022).

[114] Orru G Storari M Scano A Piras V Taibi R Viscuso D. Obstructive Sleep Apnea, oxidative stress, inflammation and endothelial dysfunction-an overview of predictive laboratory biomarkers. Eur Rev Med Pharmacol Sci. (2020) 24:6939–48. 10.26355/eurrev_202006_21685 32633387

[115] Javaheri S Barbe F Campos-Rodriguez F Dempsey JA Khayat R Javaheri S. Sleep apnea: types, mechanisms, and clinical cardiovascular consequences. J Am Coll Cardiol. (2017) 69:841–58. 10.1016/j.jacc.2016.11.069 28209226 PMC5393905

[116] Lorenzi-Filho G Almeida FR Strollo PJ. Treating OSA: current and emerging therapies beyond CPAP. Respirology. (2017) 22:1500–7. 10.1111/resp.13144 28901030

[117] Tóthová L Celec P Mucska I Hodosy J. Short-term effects of continuous positive airway pressure on oxidative stress in severe sleep apnea. Sleep Breath. (2019) 23:857–63. 10.1007/s11325-018-01777-0 30685847

[118] Halaris A Leonard BE. Preface. Mod Trends Pharmacopsychiatry. (2013) 28:VII–VIII. 10.1159/isbn.978-3-318-02311-4 25286461

[119] Celano CM Daunis DJ Lokko HN Campbell KA Huffman JC. Anxiety disorders and cardiovascular disease. Curr Psychiatry Rep. (2016) 18:101. 10.1007/s11920-016-0739-5 27671918 PMC5149447

[120] Vavakova M Durackova Z Trebaticka J. Markers of oxidative stress and neuroprogression in depression disorder. Oxid Med Cell Longev. (2015) 2015:898393. 10.1155/2015/898393 26078821 PMC4453280

[121] Juszczyk G Mikulska J Kasperek K Pietrzak D Mrozek W Herbet M. Chronic stress and oxidative stress as common factors of the pathogenesis of depression and Alzheimer's disease: the role of antioxidants in prevention and treatment. Antioxidants. (2021) 10:1439. 10.3390/antiox10091439 34573069 PMC8470444

[122] Steenkamp LR Hough CM Reus VI Jain FA Epel ES James SJ. Severity of anxiety– but not depression– is associated with oxidative stress in Major Depressive Disorder. J Affect Disord. (2017) 219:193–200. 10.1016/j.jad.2017.04.042 28564628 PMC5550320

[123] Fedoce ADG Ferreira F Bota RG Bonet-Costa V Sun PY Davies KJA. The role of oxidative stress in anxiety disorder: cause or consequence? Free Radic Res. (2018) 52:737–50. 10.1080/10715762.2018.1475733 29742940 PMC6218334

[124] Liu T Zhong S Liao X Chen J He T Lai S. A meta-analysis of oxidative stress markers in depression. PLoS One. (2015) 10:e0138904. 10.1371/journal.pone.0138904 26445247 PMC4596519

[125] Panahi D Pirposhteh EA Moradi B Poursadeqiyan M Sahlabadi AS. Effectiveness of educational intervention on reducing oxidative stress caused by occupational stress in nurses: a health promotion approach. J Educ Health Promot. (2022) 55:56. 10.4103/jehp.jehp_1425_21 PMC9621363 36325207

[126] Verdoia M Schaffer A Sartori C Barbieri L Cassetti E. Vitamin D deficiency is independently associated with the extent of coronary artery disease. Eur J Clin Invest. (2014) 44:634–42. 10.1111/eci.12281 24829065

[127] Wang Y Zhang H. Serum 25-hydroxyvitamin D3 levels are associated with carotid intima-media thickness and carotid atherosclerotic plaque in type 2 diabetic patients. J Diabetes Res. (2017) 2017:3510275. 10.1155/2017/3510275 28459072 PMC5387802

[128] Holick MF. Vitamin D status: measurement, interpretation, clinical application. Ann Epidemiol. (2009) 19:73–8. 10.1016/j.annepidem.2007.12.001 18329892 PMC2665033

[129] Lavie CJ Lee JH Milani RV. Vitamin D and cardiovascular disease will it live up to its hype? J Am Coll Cardiol. (2011) 58:1547–56. 10.1016/j.jacc.2011.07.008 21958881

[130] Wimalawansa SJ. Vitamin D deficiency: effects on oxidative stress, epigenetics, gene regulation, and aging. Biology. (2019) 8:30. 10.3390/biology8020030 31083546 PMC6627346

[131] Ricca C Aillon A Bergandi L Alotto D Castagnoli C Silvagno F. Vitamin D receptor is necessary for mitochondrial function and cell health. Int J Mol Sci. (2018) 19:1672. 10.3390/ijms19061672 29874855 PMC6032156

[132] Salamanna F Maglio M Sartori M Landini MP Fini M. Vitamin D and platelets: a menacing duo in COVID-19 and potential relation to bone remodeling. Int J Mol Sci. (2021) 22:10010. 10.3390/ijms221810010 34576172 PMC8468972

[133] Zhou W Wang W Yuan XJ Xiao CC Xing Y Ye SD. The effects of RBP4 and vitamin D on the proliferation and migration of vascular smooth muscle cells via the JAK2/STAT3 signaling pathway. Oxid Med Cell Longev. (2022) 2022:3046777. 10.1155/2022/3046777 35082965 PMC8786468

[134] Kim D-H Meza CA Clarke H Kim J-S Hickner RC. Vitamin D and endothelial function. Nutrients. (2020) 12:575. 10.3390/nu12020575 32098418 PMC7071424

[135] Cimmino G Conte S Morello M Pellegrino G Marra L Morello A. Vitamin D inhibits IL-6 pro-atherothrombotic effects in human endothelial cells: a potential mechanism for protection against COVID-19 infection? J Cardiovasc Dev Dis. (2022) 9:27. 10.3390/jcdd9010027 35050236 PMC8781542

[136] Uberti F Lattuada D Morsanuto V Nava U Bolis G Vacca G. Vitamin D protects human endothelial cells from oxidative stress through the autophagic and survival pathways. J Clin Endocrinol Metab. (2014) 99:1367–1374. 10.1210/jc.2013-2103 24285680

[137] de la Guía-Galipienso F Martínez-Ferran M Vallecillo N Lavie CJ Sanchis-Gomar F Pareja-Galeano H. Vitamin D and cardiovascular health. Clin Nutr. (2021) 40:2946–57. 10.1016/j.clnu.2020.12.025 33397599 PMC7770490

[138] Boucher BJ Grant WB. Difficulties in designing randomised controlled trials of vitamin D supplementation for reducing acute cardiovascular events and in the analysis of their outcomes. Int J Cardiol Heart Vasculat. (2020) 29:100564. 10.1016/j.ijcha.2020.100564 32617386 PMC7322678

[139] Witkowski M Weeks TL Hazen SL. Gut microbiota and cardiovascular disease. Circ Res. (2020) 127:553–70. 10.1161/CIRCRESAHA.120.316242 32762536 PMC7416843

[140] Jie Z Xia H Zhong S-L Feng Q Li S Liang S. The gut microbiome in atherosclerotic cardiovascular disease. Nat Commun. (2017) 8:845. 10.1038/s41467-017-00900-1 29018189 PMC5635030

[141] Zhu Q Gao R Zhang Y Pan D Zhu Y Zhang X. Dysbiosis signatures of gut microbiota in coronary artery disease. Physiol Genomics. (2018) 50:893–903. 10.1152/physiolgenomics.00070.2018 30192713

[142] Ascher S Reinhardt C. The gut microbiota: an emerging risk factor for cardiovascular and cerebrovascular disease. Eur J Immunol. (2018) 48:564–75. 10.1002/eji.201646879 29230812

[143] Hamer HM Jonkers DM Bast A Vanhoutvin SA Fischer MA Kodde A. Butyrate modulates oxidative stress in the colonic mucosa of healthy humans. Clin Nutr. (2009) 28:88–93. 10.1016/j.clnu.2008.11.002 19108937

[144] Valladares R Sankar D Li N Williams E Lai KK Abdelgeliel AS. Lactobacillus johnsonii N6.2 mitigates the development of type 1 diabetes in BB-DP rats. PLoS ONE. (2010) 5:e10507. 10.1371/journal.pone.0010507 20463897 PMC2865539

[145] Leschelle X Goubern M Andriamihaja M Blottière HM Couplan E Gonzalez-Barroso MD. Adaptative metabolic response of human colonic epithelial cells to the adverse effects of the luminal compound sulfide. Biochim Biophys Acta. (2005) 1725:201–12. 10.1016/j.bbagen.2005.06.002 15996823

[146] Qiao Y Sun J Ding Y Le G Shi Y. Alterations of the gut microbiota in high-fat diet mice is strongly linked to oxidative stress. Appl Microbiol Biotechnol. (2013) 97:1689–97. 10.1007/s00253-012-4323-6 22948953

[147] Xu CC Yang SF Zhu LH Cai X Sheng YS Zhu SW. Regulation of N-acetyl cysteine on gut redox status and major microbiota in weaned piglets. J Anim Sci. (2014) 92:1504–11. 10.2527/jas.2013-6755 24496840

[148] Shandilya S Kumar S Kumar Jha N Kumar Kesari K Ruokolainen J. Interplay of gut microbiota and oxidative stress: perspective on neurodegeneration and neuroprotection. J Adv Res. (2022) 38:223–44. 10.1016/j.jare.2021.09.005 35572407 PMC9091761

[149] Rahman MM Islam F Or-Rashid MH Mamun AA Rahaman MS Islam MM. The gut microbiota (microbiome) in cardiovascular disease and its therapeutic regulation. Front Cell Infect Microbiol. (2022) 12:903570. 10.3389/fcimb.2022.903570 35795187 PMC9251340

[150] Dregan A Charlton J Chowienczyk P Gulliford MC. Chronic inflammatory disorders and risk of type 2 diabetes mellitus, coronary heart disease, and stroke: a population-based cohort study. Circulation. (2014) 130:837–44. 10.1161/CIRCULATIONAHA.114.009990 24970784

[151] Crowson CS Liao KP Davis JM 3rd Solomon DH Matteson EL Knutson KL. Rheumatoid arthritis and cardiovascular disease. Am Heart J. (2013) 166:622–8.e1. 10.1016/j.ahj.2013.07.010 24093840 PMC3890244

[152] Asanuma YF. Accelerated atherosclerosis and inflammation in systemic lupus erythematosus. Nihon Rinsho Meneki Gakkai Kaishi. (2012) 35:470–80. 10.2177/jsci.35.470 23291482

[153] Smallwood MJ Nissim A Knight AR Whiteman M Haigh R Winyard PG. Oxidative stress in autoimmune rheumatic diseases. Free Radic Biol Med. (2018) 125:3–14. 10.1016/j.freeradbiomed.2018.05.086 29859343

[154] López-Armada MJ Fernández-Rodríguez JA Blanco FJ. Mitochondrial dysfunction and oxidative stress in rheumatoid arthritis. Antioxidants. (2022) 11:1151. 10.3390/antiox11061151 35740048 PMC9220001

[155] Danesh Yazdi M Wang Y Di Q Wei Y Requia WJ Shi L. Long-term association of air pollution and hospital admissions among medicare participants using a doubly robust additive model. Circulation. (2021) 143:1584–96. 10.1161/CIRCULATIONAHA.120.050252 33611922 PMC8055197

[156] Lelieveld J Klingmuller K Pozzer A Poschl U Fnais M Daiber A. Cardiovascular disease burden from ambient air pollution in Europe reassessed using novel hazard ratio functions. Eur Heart J. (2019) 40:1590–6. 10.1093/eurheartj/ehz135 30860255 PMC6528157

[157] Leni Z Künzi L Geiser M. Air pollution causing oxidative stress. Curr Opin Toxicol. (2020) 20–21:1–8. 10.1016/j.cotox.2020.02.006

[158] Hahad O Lelieveld J Birklein F Lieb K Daiber A Münzel T. Ambient air pollution increases the risk of cerebrovascular and neuropsychiatric disorders through induction of inflammation and oxidative stress. Int J Mol Sci. (2020) 21:4306. 10.3390/ijms21124306 32560306 PMC7352229

[159] Abohashem S Osborne MT Dar T Naddaf N Abbasi T Ghoneem A. A leucopoietic-arterial axis underlying the link between ambient air pollution and cardiovascular disease in humans. Eur Heart J. (2021) 42:761–72. 10.1093/eurheartj/ehaa982 33428721 PMC7882372

[160] Zhao M Hoek G Strak M Grobbee DE Graham I Klipstein-Grobusch K. A global analysis of associations between fine particle air pollution and cardiovascular risk factors: feasibility study on data linkage. Glob Heart. (2020) 15:53. 10.5334/gh.877 32923347 PMC7427684

[161] Huang C Moran AE Coxson PG Yang X Liu F Cao J. Potential cardiovascular and total mortality benefits of air pollution control in urban China. Circulation. (2017) 136:1575–84. 10.1161/CIRCULATIONAHA.116.026487 28882886 PMC5665369

[162] Sarnak MJ Levey AS Schoolwerth AC Coresh J Culleton B Hamm LL. Kidney disease as a risk factor for development of cardiovascular disease. Circulation. (2003) 108:2154–69. 10.1161/01.CIR.0000095676.90936.80 14581387

[163] Mafham M Emberson J Landray MJ Wen CP Baigent C. Estimated glomerular filtration rate and the risk of major vascular events and all-cause mortality: a meta-analysis. PLoS ONE. (2011) 6:e25920. 10.1371/journal.pone.0025920 22039429 PMC3198450

[164] van der Velde M Matsushita K Coresh J Astor BC Woodward M Levey A. Lower estimated glomerular filtration rate and higher albuminuria are associated with all-cause and cardiovascular mortality. A collaborative meta-analysis of high-risk population cohorts. Kidney Int. (2011) 79:1341–52. 10.1038/ki.2010.536 21307840

[165] Yuan J Zou X-R Han S-P Cheng H Wang L Wang J-W. Prevalence and risk factors for cardiovascular disease among chronic kidney disease patients: results from the Chinese cohort study of chronic kidney disease (C-STRIDE). BMC Nephrol. (2017) 18:23. 10.1186/s12882-017-0441-9 28088175 PMC5237491

[166] Vallianou NG Mitesh S Gkogkou A Geladari E. Chronic kidney disease and cardiovascular disease: is there any relationship? Curr Cardiol Rev. (2018) 15:55–63. 10.2174/1573403X14666180711124825 29992892 PMC6367692

[167] Umbro I Fabiani V Fabiani M Angelico F Del Ben M. A systematic review on the association between obstructive sleep apnea and chronic kidney disease. Sleep Med Rev. (2020) 53:101337. 10.1016/j.smrv.2020.101337 32629235

[168] Jean G Souberbielle J Chazot C. Vitamin D in chronic kidney disease and dialysis patients. Nutrients. (2017) 9:328. 10.3390/nu9040328 28346348 PMC5409667

[169] Chen Y Cao F Xiao J-P Fang X-Y Wang X-R Ding L-H. Emerging role of air pollution in chronic kidney disease. Environ Sci Pollut Res. (2021) 28:52610–24. 10.1007/s11356-021-16031-6 34448134

[170] Feng Z Wang T Dong S Jiang H Zhang J Raza HK. Association between gut dysbiosis and chronic kidney disease: a narrative review of the literature. J Int Med Res. (2021) 49:030006052110532. 10.1177/03000605211053276 34704483 PMC8554569

[171] Simoes e Silva AC Miranda AS Rocha NP Teixeira AL. Neuropsychiatric disorders in chronic kidney disease. Front Pharmacol. (2019) 10:932. 10.3389/fphar.2019.00932 31474869 PMC6707423

[172] Chang A Van Horn L Jacobs DR Liu K Muntner P Newsome B. Lifestyle-related factors, obesity, and incident microalbuminuria: the CARDIA (coronary artery risk development in young adults) study. Am J Kidney Dis. (2013) 62:267–75. 10.1053/j.ajkd.2013.02.363 23601954 PMC3720776

[173] Okabayashi Y Tsuboi N Sasaki T Haruhara K Kanzaki G Koike K. Glomerulopathy associated with moderate obesity. Kidney Int Rep. (2016) 1:250–5. 10.1016/j.ekir.2016.08.006 29142929 PMC5678835

[174] Minutolo R De Nicola L Mazzaglia G Postorino M Cricelli C Mantovani LG. Detection and awareness of moderate to advanced CKD by primary care practitioners: a cross-sectional study from Italy. Am J Kidney Dis. (2008) 52:444–53. 10.1053/j.ajkd.2008.03.002 18468747

[175] Chen Y-C Lin H-Y Li C-Y Lee M-S Su YC. A nationwide cohort study suggests that hepatitis C virus infection is associated with increased risk of chronic kidney disease. Kidney Int. (2014) 85:1200–7. 10.1038/ki.2013.455 24257691

[176] Gherghina ME Peride I Tiglis M Neagu TP Niculae A Checherita IA. Uric acid and oxidative stress-relationship with cardiovascular, metabolic, and renal impairment. Int J Mol Sci. (2022) 23:3188. 10.3390/ijms23063188 35328614 PMC8949471

[177] Duni A Liakopoulos V Roumeliotis S Peschos D Dounousi E. Oxidative stress in the pathogenesis and evolution of chronic kidney disease: Untangling Ariadne's thread. Int J Mol Sci. (2019) 20:3711 10.3390/ijms20153711 31362427 PMC6695865

[178] Ferrer LM Monroy AM Lopez-Pastrana J Nanayakkara G Cueto R Li Y-F. Caspase-1 plays a critical role in accelerating chronic kidney disease-promoted neointimal hyperplasia in the carotid artery. J Cardiovasc Transl Res. (2016) 9:135–144. 10.1007/s12265-016-9683-3 26928596 PMC5131710

[179] Bray G. Obesity increases risk for diabetes. Int J Obesity Relat Metab Disord. (1992) 16:S13–7. 1338382

[180] Sami W Ansari T Butt NS Ab Hamid RM. Effect of diet on type 2 diabetes mellitus: a review. Int J Health Sci. (2017) 11:65. 10.1002/dmrr.2515 28539866 PMC5426415

[181] Aune D Norat T Leitzmann M Tonstad S Vatten LJ. Physical activity and the risk of type 2 diabetes: a systematic review and dose–response meta-analysis. Eur J Epidemiol. (2015) 30:529–42. 10.1007/s10654-015-0056-z 26092138

[182] Suez J Korem T Zeevi D Zilberman-Schapira G Thaiss CA Maza O. Artificial sweeteners induce glucose intolerance by altering the gut microbiota. Nature. (2014) 514:181–6. 10.1038/nature13793 25231862

[183] Thomas C Wurzer L Malle E Ristow M Madreiter-Sokolowski CT. Modulation of reactive oxygen species homeostasis as a pleiotropic effect of commonly used drugs. Front Aging. (2022) 3:905261. 10.3389/fragi.2022.905261 35821802 PMC9261327

[184] Newman CB Preiss D Tobert JA Jacobson TA Page RL 2nd Goldstein LB. Statin safety and associated adverse events: a scientific statement from the American Heart Association. Arterioscler Thromb Vasc Biol. (2019) 39:e38–e81. 10.1161/ATV.0000000000000073 30580575

[185] Pignatelli P Carnevale R Pastori D Cangemi R Napoleone L Bartimoccia S. Immediate antioxidant and antiplatelet effect of atorvastatin via inhibition of Nox2. Circulation. (2012) 126:92–103. 10.1161/CIRCULATIONAHA.112.095554 22615342

[186] Margaritis M Sanna F Antoniades C. Statins and oxidative stress in the cardiovascular system. Curr Pharm Des. (2018) 23:7040–7. 10.2174/1381612823666170926130338 28950822

[187] Zinellu A Mangoni AA. A systematic review and meta-analysis of the effect of statins on glutathione peroxidase, superoxide dismutase, and catalase. Antioxidants. (2021) 10:1841. 10.3390/antiox10111841 34829712 PMC8614838

[188] Chen S Liu B Kong D Li S Li C Wang H. Atorvastatin calcium inhibits phenotypic modulation of PDGF-BB-induced VSMCs via down-regulation the Akt signaling pathway. PLoS ONE. (2015) 10:e0122577. 10.1371/journal.pone.0122577 25874930 PMC4398430

[189] Ferguson JF Hinkle CC Mehta NN Bagheri R Derohannessian SL Shah R. Translational studies of lipoprotein-associated phospholipase A in inflammation and atherosclerosis. J Am Coll Cardiol. (2012) 59:764–72. 10.1016/j.jacc.2011.11.019 22340269 PMC3285416

[190] White HD Simes J Stewart RAH Blankenberg S Barnes EH Marschner IC. Changes in lipoprotein-associated phospholipase A2 activity predict coronary events and partly account for the treatment effect of pravastatin: results from the long-term intervention with pravastatin in ischemic disease study. J Am Heart Assoc. (2013) 2:e000360. 10.1161/JAHA.113.000360 24152981 PMC3835245

[191] Pinkosky SL Newton RS Day EA Ford RJ Lhotak S Austin RC. Liver-specific ATP-citrate lyase inhibition by bempedoic acid decreases LDL-C and attenuates atherosclerosis. Nat Commun. (2016) 7:13457. 10.1038/ncomms13457 27892461 PMC5133702

[192] Ballantyne CM Laufs U Ray KK Leiter LA Bays HE Goldberg AC. Bempedoic acid plus ezetimibe fixed-dose combination in patients with hypercholesterolemia and high CVD risk treated with maximally tolerated statin therapy. Eur J Prev Cardiol. (2020) 27:593–603. 10.1177/2047487319864671 31357887 PMC7153222

[193] Goldberg AC Leiter LA Stroes ESG Baum SJ Hanselman JC Bloedon LT. Effect of bempedoic acid vs placebo added to maximally tolerated statins on low-density lipoprotein cholesterol in patients at high risk for cardiovascular disease. JAMA. (2019) 322:1780. 10.1001/jama.2019.16585 31714986 PMC6865290

[194] Zhao X Ma X Luo X Shi Z Deng Z Jin Y. Efficacy and safety of bempedoic acid alone or combining with other lipid-lowering therapies in hypercholesterolemic patients: a meta-analysis of randomized controlled trials. BMC Pharmacol Toxicol. (2020) 21:86. 10.1186/s40360-020-00463-w 33276805 PMC7716459

[195] Bays HE Baum SJ Brinton EA Plutzky J Hanselman JC Teng R. Effect of bempedoic acid plus ezetimibe fixed-dose combination vs ezetimibe or placebo on low-density lipoprotein cholesterol in patients with type 2 diabetes and hypercholesterolemia not treated with statins. Am J Prevent Cardiol. (2021) 8:100278. 10.1016/j.ajpc.2021.100278 34746903 PMC8550983

[196] Lloyd-Jones DM Morris PB Ballantyne CM Birtcher KK Covington AM DePalma SM. Wilkins: 2022 ACC Expert Consensus Decision Pathway on the Role of Nonstatin Therapies for LDL-Cholesterol Lowering in the Management of Atherosclerotic Cardiovascular Disease Risk: A Report of the American College of Cardiology Solution Set Oversight Committee. J Am Coll Cardiol. (2022) 80:1366–418. 10.1016/j.jacc.2022.07.006 36031461

[197] Marston NA Giugliano RP Im K Silverman MG O'Donoghue ML Wiviott SD. Association between triglyceride lowering and reduction of cardiovascular risk across multiple lipid-lowering therapeutic classes: a systematic review and meta-regression analysis of randomized controlled trials. Circulation. (2019) 140:1308–17. 10.1161/CIRCULATIONAHA.119.041998 31530008 PMC6791781

[198] Prasad A. Biochemistry and molecular biology of mechanisms of action of fibrates–an overview. Int J Biochem Res Rev. (2019) 26:1–12 10.9734/ijbcrr/2019/v26i2300942019

[199] Raslová K Nagyová A Dobiásová M Ptácková K Dusinská M. Effect of ciprofibrate on lipoprotien metabolism and oxidative stress parameters in patients with type 2 diabetes mellitus and atherogenic lipoprotein phenotype. Acta Diabetol. (2000) 37:131–4. 10.1007/s005920070015 11277313

[200] Sabatine MS Giugliano RP Wiviott SD Raal FJ Blom DJ Robinson J. Open-label study of long-term evaluation against: efficacy and safety of evolocumab in reducing lipids and cardiovascular events. N Engl J Med. (2015) 372:1500–9. 10.1056/NEJMoa1500858 25773607

[201] Robinson JG Farnier M Krempf M Bergeron J Luc G Averna M. Efficacy and safety of alirocumab in reducing lipids and cardiovascular events. N Engl J Med. (2015) 372:1489–99. 10.1056/NEJMoa1501031 25773378

[202] Schwartz GG Steg PG Szarek M Bhatt DL Bittner VA Diaz R. Alirocumab and cardiovascular outcomes after acute coronary syndrome. N Engl J Med. (2018) 379:2097–107. 10.1056/NEJMoa1801174 30403574

[203] Cammisotto V Baratta F Simeone PG Barale C Lupia E Galardo G. Proprotein convertase subtilisin kexin type 9 (PCSK9) beyond lipids: the role in oxidative stress and thrombosis. Antioxidants. (2022) 11:569. 10.3390/antiox11030569 35326219 PMC8945358

[204] Third Third report of the national cholesterol education program (NCEP) expert panel on detection evaluation and and treatment of high blood cholesterol in adults (Adult Treatment Panel III) final report. Circulation. (2002) 106:3143–421 10.1161/circ.106.25.3143 12485966

[205] Ganji SH Qin S Zhang L Kamanna VS Kashyap ML. Niacin inhibits vascular oxidative stress, redox-sensitive genes, and monocyte adhesion to human aortic endothelial cells. Atherosclerosis. (2009) 202:68–75. 10.1016/j.atherosclerosis.2008.04.044 18550065

[206] Hamoud S Kaplan M Meilin E Hassan A Torgovicky R Cohen R. Niacin administration significantly reduces oxidative stress in patients with hypercholesterolemia and low levels of high-density lipoprotein cholesterol. Am J Med Sci. (2013) 345:195–9. 10.1097/MAJ.0b013e3182548c28 22990043

[207] Romani M Hofer DC Katsyuba E Auwerx J. Niacin: an old lipid drug in a new NAD+ dress. J Lipid Res. (2019) 60:741–6. 10.1194/jlr.S092007 30782960 PMC6446705

[208] Ilkhani F Hosseini B Saedisomeolia A. Niacin and oxidative stress: a mini-review. J Nutr Med Diet Care. (2016) 2:014. 10.23937/2572-3278.151001428591759

[209] Skulas-Ray AC Wilson PWF Harris WS Brinton EA Kris-Etherton PM Richter CK. Omega-3 fatty acids for the management of hypertriglyceridemia: a science advisory from the American Heart Association. Circulation. (2019) 140:e673–91. 10.1161/CIR.0000000000000709 31422671

[210] Heshmati J Morvaridzadeh M Maroufizadeh S Akbari A Yavari M Amirinejad A. Omega-3 fatty acids supplementation and oxidative stress parameters: a systematic review and meta-analysis of clinical trials. Pharmacol Res. (2019) 149:104462. 10.1016/j.phrs.2019.104462 31563611

[211] Rahimi K Bidel Z Nazarzadeh M Copland E Canoy D Ramakrishnan R. Pharmacological blood pressure lowering for primary and secondary prevention of cardiovascular disease across different levels of blood pressure: an individual participant-level data meta-analysis. Lancet. (2021) 397:1625–36. 10.1016/S0140-6736(21)00590-0 33933205 PMC8102467

[212] Snow V Barry P Fihn SD Gibbons RJ Owens DK Williams SV. Primary care management of chronic stable angina and asymptomatic suspected or known coronary artery disease: a clinical practice guideline from the American College of Physicians. Ann Intern Med. (2004) 141:562–7. 10.7326/0003-4819-141-7-200410050-00014 15466774

[213] Vukelic S Griendling KK. Angiotensin II, from vasoconstrictor to growth factor: a paradigm shift. Circ Res. (2014) 114:754–7. 10.1161/CIRCRESAHA.114.303045 24577962 PMC3985550

[214] Hornig B Landmesser U Kohler C Ahlersmann D Spiekermann S Christoph A. Comparative effect of ACE inhibition and angiotensin II type 1 receptor antagonism on bioavailability of nitric oxide in patients with coronary artery disease. Circulation. (2001) 103:799–805. 10.1161/01.CIR.103.6.799 11171786

[215] Liang W Tan CY Ang L Sallam N Granville DJ Wright JM. Ramipril improves oxidative stress-related vascular endothelial dysfunction in db/db mice. J Physiol Sci. (2008) 58:405–11. 10.2170/physiolsci.RP012808 18845058

[216] Mason RP. Mechanisms of plaque stabilization for the dihydropyridine calcium channel blocker amlodipine: review of the evidence. Atherosclerosis. (2002) 165:191–9. 10.1016/S0021-9150(01)00729-8 12417269

[217] Kim HJ Han SJ Kim DJ Jang HC Lim S Choi SH. Effects of valsartan and amlodipine on oxidative stress in type 2 diabetic patients with hypertension: a randomized, multicenter study. Korean J Intern Med. (2017) 32:497–504. 10.3904/kjim.2015.404 28490725 PMC5432799

[218] Matsuda Y Akita H Terashima M Shiga N Kanazawa K Yokoyama M. Carvedilol improves endothelium-dependent dilatation in patients with coronary artery disease. Am Heart J. (2000) 140:753–9. 10.1067/mhj.2000.110093 11054621

[219] Kametani R Miura T Harada N Shibuya M Wang R Tan H. Carvedilol inhibits mitochondrial oxygen consumption and superoxide production during calcium overload in isolated heart mitochondria. Circ J. (2006) 70:321–6. 10.1253/circj.70.321 16501300

[220] Zepeda RJ Castillo R Rodrigo R Prieto JC Aramburu I Brugere S. Effect of carvedilol and nebivolol on oxidative stress-related parameters and endothelial function in patients with essential hypertension. Basic Clin Pharmacol Toxicol. (2012) 111:309–16. 10.1111/j.1742-7843.2012.00911.x 22703478

[221] Sudano I Osto E Ruschitzka F. Blood Pressure-Lowering Therapy. Berlin Heidelberg: Springer (2020).

[222] Yaribeygi H Atkin SL Sahebkar A. A review of the molecular mechanisms of hyperglycemia-induced free radical generation leading to oxidative stress. J Cell Physiol. (2019) 234:1300–12. 10.1002/jcp.27164 30146696

[223] Li FF Fu LY Xu XH Su XF Wu JD Ye L. Analysis of the add-on effect of α-glucosidase inhibitor, acarbose in insulin therapy: a pilot study. Biomed Rep. (2016) 5:461–6. 10.3892/br.2016.744 27699014 PMC5038828

[224] Rizzo MR Barbieri M Marfella R Paolisso G. Reduction of oxidative stress and inflammation by blunting daily acute glucose fluctuations in patients with type 2 diabetes: role of dipeptidyl peptidase-IV inhibition. Diabetes Care. (2012) 35:2076–82. 10.2337/dc12-0199 22688551 PMC3447848

[225] Del Guerra S Grupillo M Masini M Lupi R Bugliani M Torri S. Gliclazide protects human islet beta-cells from apoptosis induced by intermittent high glucose. Diabetes Metab Res Rev. (2007) 23:234–8. 10.1002/dmrr.680 16952202

[226] Owen MR Doran E Halestrap AP. Evidence that metformin exerts its anti-diabetic effects through inhibition of complex 1 of the mitochondrial respiratory chain. Biochem J. (2000) 348:607–14 10.1042/bj3480607 10839993 PMC1221104

[227] Ashabi G Khalaj L Khodagholi F Goudarzvand M Sarkaki A. Pre-treatment with metformin activates Nrf2 antioxidant pathways and inhibits inflammatory responses through induction of AMPK after transient global cerebral ischemia. Metab Brain Dis. (2015) 30:747–54. 10.1007/s11011-014-9632-2 25413451

[228] D. Diniz Vilela, Gomes Peixoto L, Teixeira RR, Belele Baptista N, Carvalho Caixeta D, Vieira de Souza A, et al. The role of metformin in controlling oxidative stress in muscle of diabetic rats. Oxid Med Cell Longev. (2016) 2016:6978625. 10.1155/2016/6978625 27579154 PMC4989083

[229] Zhu Q Ni XQ Lu WW Zhang JS Ren JL Wu D. Intermedin reduces neointima formation by regulating vascular smooth muscle cell phenotype via cAMP/PKA pathway. Atherosclerosis. (2017) 266:212–22. 10.1016/j.atherosclerosis.2017.10.011 29053988

[230] Krasner NM Ido Y Ruderman NB Cacicedo JM. Glucagon-like peptide-1 (GLP-1) analog liraglutide inhibits endothelial cell inflammation through a calcium and AMPK dependent mechanism. PLoS ONE. (2014) 9:e97554. 10.1371/journal.pone.0097554 24835252 PMC4023984

[231] Uthman L Baartscheer A Schumacher CA Fiolet JWT Kuschma MC Hollmann MW. Direct cardiac actions of sodium glucose cotransporter 2 inhibitors target pathogenic mechanisms underlying heart failure in diabetic patients. Front Physiol. (2018) 9:1575. 10.3389/fphys.2018.01575 30519189 PMC6259641

[232] Osman I Segar L. Pioglitazone, a PPARgamma agonist, attenuates PDGF-induced vascular smooth muscle cell proliferation through AMPK-dependent and AMPK-independent inhibition of mTOR/p70S6K and ERK signaling. Biochem Pharmacol. (2016) 101:54–70. 10.1016/j.bcp.2015.11.026 26643070 PMC4753090

[233] Redondo S Ruiz E Santos-Gallego CG Padilla E Tejerina T. Pioglitazone induces vascular smooth muscle cell apoptosis through a peroxisome proliferator-activated receptor-γ, transforming growth factor-β1, and a Smad2-dependent mechanism. Diabetes. (2005) 54:811–7. 10.2337/diabetes.54.3.811 15734860

[234] Mazzone T Meyer PM Feinstein SB Davidson MH Kondos GT D'Agostino RB. Effect of pioglitazone compared with glimepiride on carotid intima-media thickness in type 2 diabetes: a randomized trial. JAMA. (2006) 296:2572–81. 10.1001/jama.296.21.joc60158 17101640

[235] Nissen SE Nicholls SJ Wolski K Nesto R Kupfer S Perez A. Comparison of pioglitazone vs glimepiride on progression of coronary atherosclerosis in patients with type 2 diabetes: the periscope randomized controlled trial. JAMA. (2008) 299:1561–73. 10.1001/jama.299.13.1561 18378631

[236] Saremi A Schwenke DC Buchanan TA Hodis HN Mack WJ Banerji M. Pioglitazone slows progression of atherosclerosis in prediabetes independent of changes in cardiovascular risk factors. Arteriosc Thromb Vasc Biol. (2013) 33:393–9. 10.1161/ATVBAHA.112.300346 23175674 PMC3908828

[237] Yang HB Zhao XY Zhang JY Du YY Wang XF. Pioglitazone induces regression and stabilization of coronary atherosclerotic plaques in patients with impaired glucose tolerance. Diabet Med. (2012) 29:359–65. 10.1111/j.1464-5491.2011.03458.x 21950726

[238] Tuttolomondo A Di Raimondo D Pecoraro R Arnao V Pinto A Licata G. Atherosclerosis as an Inflammatory Disease. Curr Pharm Des. (2012) 18:4266–88. 10.2174/138161212802481237 22390643

[239] Xiang P Blanchard V Francis GA. Smooth muscle cell-macrophage interactions leading to foam cell formation in atherosclerosis: location, location, location. Front Physiol. (2022) 13:921597. 10.3389/fphys.2022.921597 35795646 PMC9251363

[240] Augé N Maupas-Schwalm FO Elbaz M Thiers J-C Waysbort A Itohara S. Role for matrix metalloproteinase-2 in oxidized low-density lipoprotein–induced activation of the sphingomyelin/ceramide pathway and smooth muscle cell proliferation. Circulation. (2004) 110:571–8. 10.1161/01.CIR.0000136995.83451.1D 15277330

[241] Wilkins JT Li RC Sniderman A Chan C Lloyd-Jones DM. Discordance between apolipoprotein B and LDL-cholesterol in young adults predicts coronary artery calcificationthe CARDIA study. J Am Coll Cardiol. (2016) 67:193–201. 10.1016/j.jacc.2015.10.055 26791067 PMC6613392

[242] Otarigho B Aballay A. Cholesterol regulates innate immunity via nuclear hormone receptor NHR-8. iScience. (2020) 23:101068. 10.1016/j.isci.2020.101068 32361270 PMC7195545

[243] Meijaard E Abrams JF Slavin JL Sheil D. Dietary fats, human nutrition and the environment: balance and sustainability. Front Nutr. (2022) 9:878644. 10.3389/fnut.2022.878644 35548568 PMC9083822

[244] Sachdeva A Cannon CP Deedwania PC Labresh KA Smith SC Jr., et al. Lipid levels in patients hospitalized with coronary artery disease: an analysis of 136,905 hospitalizations in get with the guidelines. Am Heart J. (2009) 157:111–7.e2. 10.1016/j.ahj.2008.08.010 19081406

[245] Walldius G Jungner I Aastveit AH Holme I Furberg CD Sniderman AD. The apoB/apoA-I ratio is better than the cholesterol ratios to estimate the balance between plasma proatherogenic and antiatherogenic lipoproteins and to predict coronary risk. Clin Chem Lab Med. (2004) 42:1355–63. 10.1515/CCLM.2004.254 15576296

[246] Ridker PM Everett BM Thuren T MacFadyen JG Chang WH Ballantyne C. Antiinflammatory therapy with canakinumab for atherosclerotic disease. N Engl J Med. (2017) 377:1119–31. 10.1056/NEJMoa1707914 28845751

[247] Gustafsson M Borén J. Mechanism of lipoprotein retention by the extracellular matrix. Curr Opin Lipidol. (2004) 15:505–14. 10.1097/00041433-200410000-00003 15361785

[248] Yoshida T Owens GK. Molecular determinants of vascular smooth muscle cell diversity. Circ Res. (2005) 96:280–91. 10.1161/01.RES.0000155951.62152.2e 15718508

[249] Liu R Jin Y Tang WH Qin L Zhang X Tellides G. Ten-eleven translocation-2 (TET2) is a master regulator of smooth muscle cell plasticity. Circulation. (2013) 128:2047–57. 10.1161/CIRCULATIONAHA.113.002887 24077167 PMC3899790

[250] Zeng Z Xia L Fan S Zheng J Qin J Fan X. Circular RNA CircMAP3K5 acts as a MicroRNA-22-3p sponge to promote resolution of intimal hyperplasia via TET2-mediated smooth muscle cell differentiation. Circulation. (2021) 143:354–71. 10.1161/CIRCULATIONAHA.120.049715 33207953

[251] Rask-Madsen C King GL. Proatherosclerotic mechanisms involving protein kinase C in diabetes and insulin resistance. Arterioscler Thromb Vasc Biol. (2005) 25:487–96. 10.1161/01.ATV.0000155325.41507.e0 15637306

[252] Feng X Liu P Zhou X Li M-T Li F-L Wang Z. Thromboxane A2 activates YAP/TAZ protein to induce vascular smooth muscle cell proliferation and migration. J Biol Chem. (2016) 291:18947–58. 10.1074/jbc.M116.739722 27382053 PMC5009267

[253] Rade JJ Barton BA Vasan RS Kronsberg SS Xanthakis V Keaney JF. Association of thromboxane generation with survival in aspirin users and nonusers. J Am Coll Cardiol. (2022) 80:233–50. 10.1016/j.jacc.2022.04.034 35660296 PMC12175083

[254] Patrono C Rocca B. Measurement of thromboxane biosynthesis in health and disease. Front Pharmacol. (2019) 10:1244. 10.3389/fphar.2019.01244 31736753 PMC6832017

[255] Rudijanto A. The role of vascular smooth muscle cells on the pathogenesis of atherosclerosis. Acta Med Indones. (2007) 39:86-93 17933075

[256] Williams H Mill CA Monk BA Hulin-Curtis S Johnson JL George SJ. Wnt2 and WISP-1/CCN4 induce intimal thickening via promotion of smooth muscle cell migration. Arterioscler Thromb Vasc Biol. (2016) 36:1417–24. 10.1161/ATVBAHA.116.307626 27199447

[257] Bale BF Doneen AL. A guarantee of arterial wellness: new era of cardiovascular medicine. J Clin Exp Cardiolog. (2014) 5:298. 10.4172/2155-9880.1000298

[258] Feng D Esperat MC Doneen AL Bale B Song H Green AE. 8-year outcomes of a program for early prevention of cardiovascular events: a growth-curve analysis. J Cardiovasc Nurs. (2014) 30:281–91. 10.1097/JCN.0000000000000141 24717191

[259] Madder RD Husaini M Davis AT VanOosterhout S Khan M Wohns D. Large lipid-rich coronary plaques detected by near-infrared spectroscopy at non-stented sites in the target artery identify patients likely to experience future major adverse cardiovascular events. Eur Heart J Cardiovasc Imaging. (2016) 17:393–9. 10.1093/ehjci/jev340 26800770

[260] Sun J Zhao X-Q Balu N Neradilek MB Isquith DA Yamada K. Carotid plaque lipid content and fibrous cap status predict systemic CV outcomes. The MRI Substudy in AIM-HIGH. JACC Cardiovasc Imaging. (2017) 10:241–9. 10.1016/j.jcmg.2016.06.017 28279371 PMC5347460

[261] Xing L Higuma T Wang Z Aguirre AD Mizuno K Takano M. Clinical significance of lipid-rich plaque detected by optical coherence tomography: a 4-year follow-up study. J Am Coll Cardiol. (2017) 69:2502–13. 10.1016/S0735-1097(17)34364-4 28521888

[262] Cheng HG Patel BS Martin SS Blaha M Doneen A Bale B. Effect of comprehensive cardiovascular disease risk management on longitudinal changes in carotid artery intima-media thickness in a community-based prevention clinic. Arch Med Sci. (2016) 12:728–35. 10.5114/aoms.2016.60955 27478452 PMC4947619

[263] Bartlett VL Dhruva SS Shah ND Ryan P Ross JS. Feasibility of using real-world data to replicate clinical trial evidence. JAMA Netw Open. (2019) 2:e1912869. 10.1001/jamanetworkopen.2019.12869 31596493 PMC6802419

[264] Hiramatsu K Barrett A Miyata Y. Current status, challenges, and future perspectives of real-world data and real-world evidence in Japan. Drugs Real World Outcomes. (2021) 8:459–80. 10.1007/s40801-021-00266-3 34148219 PMC8605941

[265] Wehrle K Tozzi V Braune S Roßnagel F Dikow H Paddock S. Implementation of a data control framework to ensure confidentiality, integrity, and availability of high-quality real-world data (RWD) in the NeuroTransData (NTD) registry. JAMIA Open. (2022) 5:ooac017. 10.1093/jamiaopen/ooac017 35571355 PMC9097675

[266] Willeit P Tschiderer L Allara E Reuber K Seekircher L Gao L. Carotid intima-media thickness progression as surrogate marker for cardiovascular risk. Circulation. (2020) 142:621–42. 10.1161/CIRCULATIONAHA.120.046361 32546049 PMC7115957

[267] Arbab-Zadeh A Nakano M Virmani R Fuster V. Acute coronary events. Circulation. (2012) 125:1147–56. 10.1161/CIRCULATIONAHA.111.047431 22392862 PMC3322378

[268] Baber U Mehran R Sartori S Schoos MM Sillesen H Muntendam P. Prevalence, impact, and predictive value of detecting subclinical coronary and carotid atherosclerosis in asymptomatic adults: the BioImage study. J Am Coll Cardiol. (2015) 65:1065–74. 10.1016/j.jacc.2015.01.017 25790876

[269] Nicolaides A Panayiotou AG. Screening for atherosclerotic cardiovascular risk using ultrasound. J Am Coll Cardiol. (2016) 67:1275–7. 10.1016/j.jacc.2016.01.016 26988946

[270] Doneen AL Bale BF Vigerust DJ Leimgruber PP. Cardiovascular prevention: migrating from a binary to a ternary classification. Front Cardiovasc Med. (2020) 7:92. 10.3389/fcvm.2020.00092 32528979 PMC7256212

[271] Näslund U Ng N Lundgren A Fhärm E Grönlund C Johansson H. Visualization of asymptomatic atherosclerotic disease for optimum cardiovascular prevention (VIPVIZA): a pragmatic, open-label, randomised controlled trial. Lancet. (2019) 393:133–42. 10.1016/S0140-6736(18)32818-6 30522919

[272] Sabatine MS Morrow DA Jablonski KA Rice MM Warnica JW Domanski MJ. Prognostic significance of the centers for disease control/American Heart Association high-sensitivity C-reactive protein cut points for cardiovascular and other outcomes in patients with stable coronary artery disease. Circulation. (2007) 115:1528–36. 10.1161/CIRCULATIONAHA.106.649939 17372173

[273] Bakris GL. Microalbuminuria: what is it? Why is it important? What should be done about it? J Clin Hypertens. (2001) 3:99–102 10.1111/j.1524-6175.2001.00442.x 11416691 PMC8101897

[274] Nicholls SJ Hazen SL. Myeloperoxidase and cardiovascular disease. Arterioscler Thromb Vasc Biol. (2005) 25:1102–11. 10.1161/01.ATV.0000163262.83456.6d 15790935

[275] Morrow JD. Quantification of isoprostanes as indices of oxidant stress and the risk of atherosclerosis in humans. Arteriosclerosis, Thrombosis, Vascular Biology. (2005) 25:279–86. 10.1161/01.ATV.0000152605.64964.c0 15591226

[276] Evans MK Graves Jr JL Shim RS Tishkoff SA Williams WW. Race in medicine—genetic variation, social categories, and paths to health equity. New Engl J Med. (2021) 385:e45. 10.1056/NEJMp2113749 34528770

[277] Wilkins CH Schindler SE Morris JC. Addressing health disparities among minority populations. JAMA Neurol. (2020) 77:1063. 10.1001/jamaneurol.2020.1614 32539100 PMC7983552

